# Supplementary material for: Perspectives of health workers on the referral of women with obstetric complications: a qualitative study in rural Sierra Leone
Source: BMJ Open. 2020 Dec 10;10(12):e041746. doi: 10.1136/bmjopen-2020-041746 (PMC7733167; doi:10.1136/bmjopen-2020-041746)
Supplement: Supplementary data [file bmjopen-2020-041746supp002.pdf]

SUPPLEMENTARY FILE 2

Signal functions of BEmONC and CEmONC

Basic Emergency Obstetric and Neonatal Care (BEmONC)

1. Parenteral treatment of infections (antibiotics)
2. Parenteral treatment of severe pre-eclampsia/eclampsia (e.g., MgSO4)
3. Treatment of PPH (e.g., uterotonics)
4. Manual vacuum aspiration of retained products of conception
5. Assisted vaginal delivery (e.g., vacuum-assisted delivery)
6. Manual removal of placenta
7. New-born resuscitation

Comprehensive Emergency Obstetric and Neonatal Care (CEmONC)

All components of BEmONC, plus

1. Surgical capability, including anaesthesia (e.g., Caesarean Section)
2. Blood transfusion
