# Supplementary material for: Perspectives of health workers on the referral of women with obstetric complications: a qualitative study in rural Sierra Leone
Source: BMJ Open. 2020 Dec 10;10(12):e041746. doi: 10.1136/bmjopen-2020-041746 (PMC7733167; doi:10.1136/bmjopen-2020-041746)
Supplement: Supplementary data [file bmjopen-2020-041746supp003.pdf]

SUPPLEMENTARY FILE 3

Interview Guide Obstetric Referral System Northern Tonkolili District

General

1. Describe the catchment area of this health facility (number of villages, terrain).
2. Describe this health facility (number and competency of staff, average number of births and referrals).
3. Describe the referral options of this health facility (nearest health facility, nearest CHC, nearest district hospital).

Authorisation

4. Which of the staff is allowed to decide to refer women to another facility?
5. Is there always staff present that is allowed to independently refer women?
6. Does the CHO or midwife in the CHC have to be contacted before referring a woman to another facility?

Referral Process

7. Describe the steps taken when referring a pregnant or post-partum women.
8. Describe how/if referral notes are used in this health facility.
9. Describe the benefit of referral notes.
10. Describe how/if feedback is received from the referral facility.
11. Describe how/if advice is obtained from other health workers before referral.
12. Describe how/if national guidelines are used for referring a woman.
13. Describe how/if maternity patients without an emergency indication are referred to a different health facility.

Accessibility

14. Describe the accessibility of Magburaka Government Hospital from here.
15. Describe the accessibility of Masanga Hospital from here.
16. If you were to refer 10 women to a hospital, how many of them do you think will actually arrive at the hospital?
17. Describe some reasons why referred women sometimes refuse referral.
18. Describe transportation options available for referral.

Ambulance Referrals

19. Describe the process of ordering an ambulance from Magburaka Government Hospital.
20. Describe the process of ordering an ambulance from Masanga Hospital.
21. How many times have you ordered an ambulance in the past month?
22. How long does it take for the ambulance to arrive at your health facility after you have ordered the ambulance? (from Magburaka/Masanga)
23. Which of the staff is allowed to order an ambulance?
24. Is there always staff present that is allowed to order an ambulance?

25. Does the CHO or midwife in the CHC have to be contacted before ordering an ambulance?
26. Is transferral by ambulance free for the woman?
27. Describe problems with the current ambulance referral system.
28. Describe recommendations for improving the ambulance referral system.

ANC/under 5

29. Describe the antenatal care in this health facility (frequency of ANC-days, number of women)
30. Describe how/if governmental Maternity Record Cards are used during ANC visits, delivery and postnatal checks.

Masanga Hospital

31. Describe recommendations for improving the health care at Masanga Hospital.
32. Is Masanga Hospital an official referral hospital in Tonkolili District?
33. Is healthcare free for pregnant and lactating women and children under 5 at Masanga Hospital?
