# Supplementary material for: Perspectives of health workers on the referral of women with obstetric complications: a qualitative study in rural Sierra Leone
Source: BMJ Open. 2020 Dec 10;10(12):e041746. doi: 10.1136/bmjopen-2020-041746 (PMC7733167; doi:10.1136/bmjopen-2020-041746)
Supplement: Supplementary data [file bmjopen-2020-041746supp004.pdf]

SUPPLEMENTARY FILE 4

Original study protocol

Includes protocol regarding quantitative research which is not included in submitted article

Proos R, *et al. BMJ Open* 2020; 10:e041746. doi: 10.1136/bmjopen-2020-041746

The current situation of maternal health in Tonkolili District and the perceptions of health workers at peripheral health units in the catchment area of Masanga Hospital concerning the current referral system of obstetric patients

Lead applicant

Ryan Proos, medical student Leiden University Medical Center, Masanga Hospital Sierra Leone

Correspondence address: e-mail: rmjproos@gmail.com

Supervisor

Dr. T. van den Akker, Leiden University Medical Center, Netherlands (Gynaecology and Obstetrics)

Research team

Hanna Mathéron, MD Global Health and Tropical Medicine, Medical Officer Masanga Hospital

Background

Sierra Leone is one of the most dangerous countries for women to be pregnant and to give birth. According to the Sierra Leone Demographic Health Survey (2013)[1], Sierra Leone has a maternal mortality ratio (MMR) of 1,165 per 100,000 live births, the highest in the world and more than six times the global average. The UN Sustainable Development Goals (SDGs), adopted in 2015, aim for a reduction of the global maternal mortality ratio to less than 70 per 100,000 live births in 2030. In line with these SDGs, the Ministry of Health and Sanitation of Sierra Leone launched the Reproductive, Maternal, Newborn, Child and Adolescent Health (RMNCAH) Strategy in 2017, which aims for a reduction of preventable deaths of women, children and adolescents and ensuring their well-being[2]. The target is to reduce the MMR of 1,165 per 100.000 live births to 650 per 100.000 live births by 2021. With launching this strategy, the government acknowledges the need for change, but there is still a long way to go to achieve this goal.

Masanga Hospital is situated in Tonkolili district, centrally located in the Northern Province of Sierra Leone. The catchment area of Masanga Hospital comprises of approximately 150,000 inhabitants and 15 peripheral health units (PHUs). Besides Masanga Hospital there are two other hospitals in Tonkolili district, Magburaka Hospital and Lion Heart Hospital in Yele.

Currently, there is no complete and trustworthy overview of maternal health in Tonkolili District. Therefore, a baseline assessment using the WHO Reproductive Health Indicators and the RMNCAH coverage targets is needed to work towards achieving the goal set by the Ministry.

It is hypothesized that the poor referral system is a major causative factor of the current poor maternal health situation in Tonkolili district. Perceptions of health workers at PHUs concerning the referral system of obstetric patients is necessary to identify areas of improvement.

Aims and Objectives

The aim of this research project is to do a retrospective, quantitative descriptive analysis of data collected during project to describe maternal health demography, geography, delivery and complication rates of Tonkolili district, especially in relationship to the catchment area of Masanga Hospital. Additionally, an assessment of health workers’ perceptions of the current referral system of obstetric patients will be undertaken.

Proos R, *et al. BMJ Open* 2020; 10:e041746. doi: 10.1136/bmjopen-2020-041746

Research Questions

1. What is the current maternal health situation in Tonkolili District, Sierra Leone, in terms of WHO Reproductive Health Indicators and the RMNCAH coverage target matches?

The baseline assessment shall provide an overview of several WHO Reproductive Health Indicators and RMNCAH coverage targets in Tonkolili district over the period 2016-2018**.** Data collection will be done in the Peripheral Health Units (PHUs) and the district hospitals (Masanga Hospital, Magburaka Hospital and Lion Heart Hospital, Yele), in collaboration with the District Health Sister (DHS1), District Medical Officer (DMO) and the District Health Information System (DHIS).

The following data will be collected per health facility:

- Level of service provision (MCHP, CHP, CHC, District Hospital)
- Location in the catchment area
- Number of deliveries (spontaneous, assisted, CS)
- Number of live births
- Number of still births
- Number of maternal deaths
- Number of complications (antepartum haemorrhage (APH), postpartum haemorrhage (PPH), pregnancy-induced hypertension (PIH), pre-eclampsia, eclampsia, obstructed labour, post-partum sepsis)
- Number of referrals, reasons for referral
- Number of antenatal care visits
- Number of intermittent preventive treatment (IPT) during ANC

2. What are the perspectives of health workers at peripheral health units in the catchment area of Masanga Hospital concerning the current referral system of women with obstetric complications?

Perspectives of health workers working in PHUs in the catchment area of Masanga Hospital will be explored in several interviews.

The discussions will focus on the following topics:

- Communication between health centres
- Determinants of referral decision
- Actions taken before and during referral
- Effect of antenatal care on referrals
- Referral constraints

Study Design

This research project is a retrospective, quantitative descriptive analysis. Additionally, a qualitative analysis will be done using interviews. Data collection will be done in collaboration with the District Health Sister (DHS1), District Medical Officer (DMO) and the DHIS.

Patients/Participants

All women that visited a PHU or hospital in Tonkolili district for antenatal care visits, health facility deliveries or pregnancy or delivery complications during the period 2016-2018 will be included in the baseline overview. Selection bias must be considered as not all pregnant women visit a health facility. Reproductive health data is available through the centralized District Health Information System. Access to the District Health Information System will be officially requested. Data is available of approximately 12,000 facility-based live births in the district per year and therefore roughly 36,000 facility-based live births in the specified period. However, it is estimated that 46% of pregnant women in Sierra Leone give birth at home[2]. There is therefore no data available of these homebirths, but these women might have visited a health facility for antenatal care or for post-partum complications.

Health workers at PHUs in the catchment area of Masanga Hospital will be asked to participate in the interviews focusing on their perceptions of the current obstetric referral system. Written informed consent will be obtained using the attached form. An interview guide will be used.

Statistical Methods

After centralization of the data in SPSS, a baseline overview of the variables named for research question 1 will be made for each PHU and hospital and for Tonkolili district. The qualitative data of the group discussions will be analyzed using systematic text condensation.

Proos R, *et al. BMJ Open* 2020; 10:e041746. doi: 10.1136/bmjopen-2020-041746

Results

The baseline assessment shall provide an overview of several WHO Reproductive Health Indicators and the RMNCAH coverage targets in Tonkolili district over the period 2016-2018. Regional differences in maternal health outcomes in Tonkolili District will be identified. These regional differences will be linked to the accessibility of emergency obstetric care in those regions. This baseline overview will provide a basis for further research and implementation of new interventions, aimed at reaching the goal set by the Ministry.

The perceptions of health workers at PHUs in the catchment area of Masanga Hospital will illuminate constraints of the current referral system of obstetric patients. Additionally, the quality of communication between different levels of health facilities concerning referrals will be described. Possible solutions as suggested by the health workers will be described.

The results will be summarized in a descriptive article.

Ethical Considerations

Ethical Approval

Ethical approval will be obtained from the Sierra Leone Ethics and Scientific Review Committee.

Selection of study population and recruitment of research participants

All patients registered at PHUs and hospitals in Tonkolili District for antenatal care visits, deliveries, and pregnancy complications in the period 2016-2018 are selected and included. Patients are free to refuse participation. This refutation must be apparent from the District Health Information System.

Informed consent process

Receiving medical care at a PHU or hospital implicitly indicates given consent to use of registered data for medical research through the District Health Information System. Written informed consent will be obtained from participants in the group discussions. The informed consent form is included in this application.

Risks of participation

Participation in the baseline overview exposes patients to minimal chance of harm. Participation does not include any clinical intervention. Confidentiality and anonymity of patient data is maximally safeguarded.

Participation in the group discussions exposes the health workers to minimal chance of harm. Confidentiality and anonymity of participant data is maximally safeguarded.

Inducements, financial benefits, and financial costs

Participation is not rewarded with any financial, material or healthcare service benefit.

Protection of research participants' privacy and confidentiality

Research participant data is centrally stored in the District Health Information System. This digital system is password protected. To ensure confidentiality, this data is only accessible by the head researcher and supervisors.

Group discussion participant data is stored on password-protected computers in Masanga Hospital. Only the head researcher has access to the names, functions and health facilities of the participants. Reported data is anonymous and cannot be traced back to the specific health facility.

Independence of research and conflicts of interests

Research is conducted on behalf of Masanga Hospital and in collaboration with Tonkolili District. No other parties are involved. The researchers certify that they have no conflicts of interests.

Schedule

1-8 weeks:

- Contact with DMO and DHS, attending the district meetings and the district maternal mortality meetings in Magburaka
- Application for funding
- Application for District Health Information System access

8-20 weeks

- Analysation of DHIS data
- Group discussions

20-28 weeks

- Analysation and illustration of DHIS data
- Writing descriptive article

GANTT chart

	**W1-4**	**W5-8**	**W9-12**	**W13-16**	**W17-20**	**W20-24**	**W24-28**
**DMO/DHS contact**	X						
**Initiation visits in PHUs/Hospitals**	X	X					
**DHIS access application**	X	X					
**Group discussions**			X	X	X		
**Data analysis**			X	X	X	X	X
**Writing**					X	X	X
**Submitting article**							X

References

1. Sierra Leone Demographic and Health Survey 2013 via the DHS Program STATcompiler. (http://www.statcompiler.com).
2. Sierra Leone Reproductive, Maternal, Newborn, Child and Adolescent Health (RMNCAH) strategy 2017-2021, Ministry of Health Sierra Leone

Participant Informed Consent Form

Study title: Perceptions of health workers at peripheral health units in the catchment area of Masanga Hospital concerning the current referral system of obstetric patients

Researcher: Ryan Proos E-mail: ryanproos9@gmail.com

Institute: Masanga Medical Research Unit, Sierra Leone

	Add your initials if you agree
I agree that this interview will be recorded and that the data will be analyzed and reported anonymously.	
I understand that I can withdraw from this study at any time, without given reason and all my data will be deleted.	
I understand I can contact the Sierra Leone Ethics and Scientific Review Committee at any time (contact details below*).	

Name of participant: ……………………………………………………………………………………..

Signature: ……………………………………………… Date: …………………………………

Name of person taking consent: …………………………………………………………………

Signature: ……………………………………………… Date: …………………………………

* Contact details Sierra Leone Ethics and Scientific Review Committee:

E-mail: efoday@health.gov.sl

** this form needs to be signed and dated in presence of the patient. If preferred, the patient will receive a copy of this form. The forms will be stored in a secure location, ensuring patients privacy.
