# Supplementary material for: Perspectives of health workers on the referral of women with obstetric complications: a qualitative study in rural Sierra Leone
Source: BMJ Open. 2020 Dec 10;10(12):e041746. doi: 10.1136/bmjopen-2020-041746 (PMC7733167; doi:10.1136/bmjopen-2020-041746)
Supplement: Author's manuscript [file bmjopen-2020-041746.draft_revisions.pdf]

BMJ Open is committed to open peer review. As part of this commitment we make the peer review history of every article we publish publicly available.

When an article is published we post the peer reviewers' comments and the authors' responses online. We also post the versions of the paper that were used during peer review. These are the versions that the peer review comments apply to.

The versions of the paper that follow are the versions that were submitted during the peer review process. They are not the versions of record or the final published versions. They should not be cited or distributed as the published version of this manuscript.

BMJ Open is an open access journal and the full, final, typeset and author-corrected version of record of the manuscript is available on our site with no access controls, subscription charges or pay-per-view fees (http://bmjopen.bmj.com).

If you have any questions on BMJ Open's open peer review process please email info.bmjopen@bmj.com

BMJ Open

Perceptions of health workers on the referral of women with obstetric complications: a qualitative study in rural Sierra Leone

Journal:	*BMJ Open*
Manuscript ID	bmjopen-2020-041746
Article Type:	Original research
Date Submitted by the Author:	18-Jun-2020
Complete List of Authors:	Proos, Ryan; Masanga Medical Research Unit<br>Matheron, Hanna; Masanga Medical Research Unit<br>Vas Nunes, Jonathan; Masanga Medical Research Unit<br>Falama, Abdul; Tonkolili District Health Management<br>Sery Kamal, Patricia<br>Grobusch, Martin; Amsterdam University Medical Centres, Center of Tropical Medicine and Travel Medicine, Department of Infectious Diseases<br>van den Akker, Thomas; Leiden Universitair Medisch Centrum, Obstetrics and Gynaecology
Keywords:	Maternal medicine < OBSTETRICS, PUBLIC HEALTH, Organisation of health services < HEALTH SERVICES ADMINISTRATION & MANAGEMENT

SCHOLARONE™
Manuscripts

I, the Submitting Author has the right to grant and does grant on behalf of all authors of the Work (as defined in the below author licence), an exclusive licence and/or a non-exclusive licence for contributions from authors who are: i) UK Crown employees; ii) where BMJ has agreed a CC-BY licence shall apply, and/or iii) in accordance with the terms applicable for US Federal Government officers or employees acting as part of their official duties; on a worldwide, perpetual, irrevocable, royalty-free basis to BMJ Publishing Group Ltd ("BMJ") its licensees and where the relevant Journal is co-owned by BMJ to the co-owners of the Journal, to publish the Work in this journal and any other BMJ products and to exploit all rights, as set out in our licence.

The Submitting Author accepts and understands that any supply made under these terms is made by BMJ to the Submitting Author unless you are acting as an employee on behalf of your employer or a postgraduate student of an affiliated institution which is paying any applicable article publishing charge ("APC") for Open Access articles. Where the Submitting Author wishes to make the Work available on an Open Access basis (and intends to pay the relevant APC), the terms of reuse of such Open Access shall be governed by a Creative Commons licence – details of these licences and which Creative Commons licence will apply to this Work are set out in our licence referred to above.

Other than as permitted in any relevant BMJ Author's Self Archiving Policies, I confirm this Work has not been accepted for publication elsewhere, is not being considered for publication elsewhere and does not duplicate material already published. I confirm all authors consent to publication of this Work and authorise the granting of this licence.

For peer review only

Perceptions of health workers on the referral of women with obstetric complications: a qualitative study in rural Sierra Leone

Ryan Proos[1,3], Hanna Mathéron[1,2,4], Jonathan Vas Nunes[1,2,4], Abdul Falama[5], Patricia Sery Kamal[5], Martin Peter Grobusch[1,4*], Thomas van den Akker[3,6]

[1]Masanga Medical Research Unit, Masanga, Sierra Leone

[2]Masanga Hospital, Masanga, Sierra Leone

[3]Obstetrics and Gynaecology Department, Leiden University Medical Center, Leiden, The Netherlands

[4]Center for Tropical Medicine and Travel Medicine, Department of Infectious Diseases, Amsterdam University Medical Centers location AMC, Amsterdam Public Health, Amsterdam Infection & Immunity, Amsterdam, The Netherlands

[5]Tonkolili District Health Management Team, Magburaka, Sierra Leone

[6]Athena Institute, VU University, Amsterdam, The Netherlands

* Corresponding author: Prof. Dr. Martin Peter Grobusch, AUMC location AMC, Meibergdreef 9, 1105 AZ Amsterdam, The Netherlands; email: m.p.grobusch@amsterdamumc.nl

Word count: 3,999 words

For peer review only - http://bmjopen.bmj.com/site/about/guidelines.xhtml

ABSTRACT

Objectives

Sierra Leone has one of the highest maternal mortality ratios in the world. Timely and well-coordinated referrals are necessary to reduce delays in providing adequate care for women with obstetric complications. This study describes the perspectives of health workers in rural facilities in Sierra Leone concerning the referral of women with obstetric complications.

Design

A qualitative research with semi-structured interviews using open-ended questions. Data were analysed by systematic text condensation.

Setting

Interviews were held with health workers in nine peripheral health units in rural Sierra Leone.

Participants

19 health workers participated in nine interviews.

Results

From the interviews, four major themes emerged: 1) communication between health care workers; 2) underlying influences on decision-making; 3) women's compliance to referral; 4) logistic constraints.

Several factors in rural Sierra Leone are perceived to complicate timely and adequate referral of women in need of emergency obstetric care. Notable among these factors are fear among women for being referred and fear among health care workers for having

For peer review only

maternal deaths or severe obstetric complications occur at their own facilities. Furthermore, decision-making of health care workers concerning referral is negatively influenced by a hierarchical culture with high power distance between health care workers.

Conclusion

Factors identified that complicate timely and adequate referral of women in need of emergency obstetric care must be considered in efforts to reduce maternal mortality. Possible interventions that may reduce delay in referral include increased communication by mobile phones between health workers for advice and feedback regarding referrals, and involvement of influential stakeholders to increase women's compliance to referral.

ARTICLE SUMMARY

Strengths and limitations of this study

- A strength of this study is the purposive and homogenous sampling used for the selection of peripheral health units and health workers for the interviews, which is representative of health facilities and health workers in rural Sierra Leone.
- A strength of this study is the use of open-ended questions alongside an interview guide, ensuring specific topics were discussed while allowing participants to introduce and discuss additional topics.
- A limitation of this study is that the data collected using semi-structured interviews were not triangulated with quantitative data collected using other methods.
- A limitation of this study is that a limited number of interviews were conducted with a limited number of participants.

For peer review only

63 **KEYWORDS**

64 health worker; maternal health; obstetric complications; qualitative study; referral; Sierra

65 Leone

For peer review only

For peer review only - http://bmjopen.bmj.com/site/about/guidelines.xhtml

BACKGROUND

Sierra Leone has one of the highest maternal mortality ratios (MMR) in the world. According to the Sierra Leone Demographic Health survey in 2013, the MMR was 1,165 per 100,000 live births [1]. The need to reduce this extremely high ratio is evident. In order to work towards meeting the United Nations Sustainable Development Goal target of an MMR below 70 per 100 000 [2], the Ministry of Health and Sanitation of Sierra Leone launched the Reproductive, Maternal, Newborn, Child and Adolescent Health Strategy in 2017, aiming to reduce the MMR of 1,165 to 650 by 2021 [3].

An underlying factor of the high MMR in Sierra Leone is the persistent low rate of facility-based births [1]. Country-wide in 2013, only slightly more than half of the women gave birth in a health facility; 49.7% of women in rural areas versus 68.1% in urban areas [1]. The West-African Ebola outbreak from 2013 to 2016 led to a further reduction in facility-based births as a consequence of reduced possibilities and fear to access the health system during the crisis [4, 5].

Pregnant women in Sierra Leone face many barriers to facility-based birth, including long distances, inability to afford costs of transport and healthcare and lack of trust in health facilities [6]. These factors contribute to a phase-1 delay in deciding to seek healthcare in case of an emergency obstetric complication, as described in the three-phase delay model by Thaddeus and Maine [7]. Even after having decided to seek health care, women are often confronted with delays in phase 2 (transport delay), and phase 3 (delay in diagnosis and treatment at the facility) [8]. Timely and well-coordinated referrals are necessary to reduce delay in receiving adequate obstetric care for women with obstetric complications.

For peer review only - http://bmjopen.bmj.com/site/about/guidelines.xhtml

Currently, there is no literature available analysing aspects of reasons for delay of care within the obstetric referral system in rural Sierra Leone.

This study describes the perspectives of health workers in rural facilities concerning the referral system for women with obstetric complications. This data will provide better understanding of challenges within the health system faced by women who are referred with emergency obstetric complications in rural Sierra Leone.

METHODS

Study design and setting

This qualitative study following the COREQ checklist of reporting [9] (Supplementary File 1) was conducted between the 1st of September 2018 and the 15th of March 2019 in Tonkolili District. This district is located in the Northern Province of Sierra Leone and sub-divided into eleven chiefdoms. Three hospitals with Comprehensive Emergency Obstetric and Neonatal Care are located in Tonkolili District. Basic and Comprehensive Emergency Obstetric and Neonatal Care (BEmONC and CEmONC) are services fundamental to provide adequate health care during pregnancy and childbirth. The signal functions of BEmONC and CEmONC centres are summarised in Supplementary File 2. Three chiefdoms in the north of Tonkolili District, Kafe Simiria, Kalansogoia and Sambaya Bendugu, with a combined population of 113,521 (2018), are served by two CEmONC centres, Magburaka Government Hospital and Masanga Hospital, an NGO-supported government hospital. Besides the two CEmONC centres, these chiefdoms are served by fifteen peripheral health units (PHUs), including four BEmONC centres. The maternity services provided at each facility level in these chiefdoms, according to the Ministry of Health and Sanitation, are summarised in Table 1.

For peer review only - http://bmjopen.bmj.com/site/about/guidelines.xhtml

Table 1: Maternity services purportedly provided at each level of health facility

Maternal and Child Health Post (MCHP)	• Antenatal care<br>o Nutritional supplementation in pregnancy (e.g. iron, folic acid and multivitamins)<br>o Risk selection and ensuing referral<br>o Malaria intermittent preventive treatment<br>• Intra- and postpartum care<br>o Monitoring of labour by using the partograph<br>o Cord clamping<br>o Active management of the third stage of labour<br>• Postnatal care<br>o Clinical assessment of the neonate (e.g. fever, convulsions, feeding)<br>o Exclusive breastfeeding recommendation<br>o Cord care<br>o Clinical assessment of mother (e.g. temperature, blood pressure, bleeding)<br>o Family planning counselling
Community Health Post (CHP)	• MCHP services (see above)
Community Health Centre (CHC)	• MCHP services (see above)<br>*plus*<br>• Maternal anaemia and urine sediment assessment

For peer review only

	• BEmONC services (see supplementary file 2)
District Hospital	• MCHP services (see above)<br>*plus*<br>• Maternal anaemia, urine, HIV, malaria and tuberculosis assessment<br>• Ultrasound scan<br>• CEmONC services (see supplementary file 2)

However, not all facilities designated to provide BEmONC and CEmONC in rural Sierra Leone are able to provide the full range of signal functions [10, 11]. Therefore, several BEmONC and CEmONC signal functions might not actually be provided in practice. Referral occurs both in consecutive order starting from Maternal and Child Health Posts (MCHPs) as well as between lower level health facilities and district hospitals. Sierra Leone has national protocols for emergency obstetric care, including referral indications [12].

The chiefdoms in northern Tonkolili District were selected, since these three combined comprise the catchment area for emergency obstetric complications belonging to Masanga Hospital, for reasons of geography such as impassable rivers and mountains and accessibility by road.

Study Subject and Public involvement

The District Health Management Team was involved in the design, conduct, reporting and dissemination planning of our research.

Data collection

For peer review only

124 The qualitative data used in this study was collected using nine semi-structured interviews
125 conducted by RP (Master of Medicine student, male) between November 2018 and January
126 2019. Purposive sampling was used to ensure variation between selected facilities where
127 interviews were conducted. Selected facilities were thus located in different chiefdoms,
128 provided different levels of care, and had varying accessibilities of the nearest district
129 hospital (Table 2).

Table 2: Facilities where interviews were conducted

Town	Level	Distance to district hospital (km)	Travel time to district hospital* (min)
Chiefdom Kafe Simiria			
Mabontor	CHC	18.9	40
Masumbrie	CHC	21.5	40
Makontande	MCHP	28.9	50
Chiefdom Kalansogoia			
Bumbuna	CHC	42.6	70
Kamasaypana	MCHP	50.0	100
Kemedugu	MCHP	58.5	110
Chiefdom Sambaya Bendugu			
Bendugu	CHC	81.4	150
Kunya	CHP	92.3	180
Dankawalia	MCHP	71.9	135

For peer review only

* Travel time by motorbike during dry season (November – May). Travel time during rainy season (June – October) will be substantially longer. Travel time by ambulance will be shorter. Roads were unpaved.

Selected facilities were approached either by telephone calls to the in-charge health worker or by face-to-face visits to that health facility. Homogenous sampling was used as each interview was conducted with health workers of different cadres currently working in the same health centre. In total, nineteen health workers participated in the nine interviews. All participants were explained the relevance and goals of the research. No facility or individual health worker refused participation. Respective health worker cadres and competencies of the participants are summarised in Table 3.

Table 3: Cadres and competencies of respondents

Health worker (number interviewed)	Competencies
Maternal and Child Health aid (MCH aid) (10)	2 years training. Competent in basic obstetric care.
State Enrolled Clinical Health Nurse (SECHN) (2)	2,5 years training. Competent in basic obstetric care.
Community Health Assistant (CHA) (3)	2 years theoretical + 1-year practical training. Competent in basic obstetric care.
Community Health Officer (CHO) (2)	3 years theoretical + 1-year practical training. Competent in basic obstetric care. No training in emergency obstetric care.

For peer review only

For peer review only - http://bmjopen.bmj.com/site/about/guidelines.xhtml

Midwife (2)	SECHN training + 1,5 year-midwifery training. Competent in emergency obstetric care including oxytocin administration, manual placenta removal, first treatment for (pre)eclampsia. No training in vacuum extraction.

The interview guide (Supplementary File 3) was used as a framework of themes to be discussed during the interviews. The guide was initially developed using themes described by Thaddeus and Maine [7], previous literature concerning pregnancy and childbirth in Sierra Leone [6, 13], and preliminary discussions with stakeholders such as medical officers, community health officers (CHO), midwives and logistical officers employed at district referral hospitals. The guide was piloted in one PHU with two health workers. The interviews were held inside the respective health facilities where only participants and interviewer were present. The interviews were conducted in English, using open-ended questions, and lasted between 30 and 60 minutes each. Data was collected using audio recording. Repeat interviews were not carried out and transcripts were not returned to participants for comments and correction for logistical reasons: there was no funding or practical possibility to re-visit these widely spread-out facilities.

Analysis

All audio recordings of interviews were transcribed verbatim by RP using Express Scribe Transcription Software (NCH Software, Greenwood Village, Colorado, USA). Content analysis was performed by RP through systematic text condensation as described by Malterud [14]. Preliminary themes were identified after transcription of the interviews, and were further specified by systematic coding and categorisation of quotes, whereupon a condensate of every theme was written based on the quotes. Data analysis was performed manually.

For peer review only

Ethical Considerations

The study proposal was endorsed by the Masanga Medical Research Unit Scientific Review Committee. Ethical approval was obtained from the Sierra Leone National Ethics and Scientific Review Committee on 29 January 2019. Permission to conduct the study in Tonkolili District was obtained from the District Health Management Team. Written informed consent was obtained from interview participants.

RESULTS

From these interviews, four major themes emerged: 1) communication between health care workers, 2) underlying influences on decision making, 3) women's compliance to referral, 4) logistic constraints.

1) Importance of communication between staff of different health centres

Giving and receiving advice surrounding referrals. Most health workers mentioned the necessity of asking for advice when having to decide whether to continue management or refer the woman to a higher-level health centre. One community health assistant (CHA) explains:

> *"I am not saying 100% I know what I am doing. I know myself. I learn, I can just know my own area and then there are people who know better. I am just a community health officer, assistant in fact."* (male CHA, MCHP)

Advice was often asked for and given in mobile phone conversations. However, sometimes higher cadre health workers travelled to health facilities to review women themselves before advice was given. Those asked for advice include CHOs, midwives, the District Health Sisters (supervising midwives, members of the Tonkolili District Health Management Team),

For peer review only

the head of a maternity ward or a medical officer at a district hospital. However, one maternal and child health aid (MCH aid) mentioned that if she recognized a woman requiring urgent referral to a higher-level facility, she would not delay by first calling for advice but rather directly refer the woman to the community health centre (CHC). She would not, however, directly refer the woman to a district hospital or inform the CHC that this woman likely needed onward referral, since she believed that this decision had to be made by CHC staff.

Besides needing advice on whether or not to refer the woman to a higher-level facility, participants also mentioned a need for advice over the phone regarding clinical management while waiting for the ambulance to arrive, since this could take up to several hours.

Feedback after referral. Many health workers indicated that they were interested in the clinical course after a woman had been referred to the district hospital, as illustrated by one CHO:

> *"We are highly interested in feedback, because they are lives and when we call on you people to rescue, then we have interest over them."* (male CHA, CHC)

Health workers in lower level facilities expressed a specific interest in knowing the clinical management including decisions on mode of birth at the district hospital. These health workers were often approached by relatives of the referred woman requesting updates on her clinical condition and outcome. Health workers regularly marked their phone numbers on the referral notes in order to receive feedback. However, many respondents complained never receiving a response from district hospital staff. Instead, they felt forced to call the

For peer review only

district hospital themselves, and indicated this comprised a communication barrier, since it required them to spend their own mobile phone credits.

Similarly, health workers complained of never receiving discharge notes with follow-up information after the woman had been discharged from the district hospital. They generally relied on the information the woman could give them verbally.

2) Underlying influences on decision-making

Referral, perceived as the safest option for health workers. The necessity of referral, as expressed by one health worker, was often to avoid complications and maternal death occurring at their own facility.

> *"If a maternal death is here, we are going to suffer."* (female SECHN, MCHP)

Often, a referred woman was described as 'not my case'. Another health worker mentioned that when a woman was referred to a CHC, it was up to that facility to manage the woman with the complication and decide what to do. Such transfer of responsibility after referral was further illustrated by a story recounted by an MCH aid about a woman she had recently referred:

> *"Yes, I delivered her. Male baby. Fresh still birth. So it is not my problem, because I have already referred her."* (female MCH aid, MCHP)

Endangering behaviour by women requiring referral. Some women did not want to be referred and health workers were under a lot of pressure from women and relatives while making a referral decision. Women and relatives were at times perceived not to tell the truth when questioned about history, since they wanted to prevent referral. One health worker mentioned that he sometimes heard rumours in the community that the point in

For peer review only

time a woman and her relatives indicated as when onset of symptoms occurred was not always correct. Most respondents described that when a woman and her relatives were told that she needed to be referred, they started begging health workers not to refer her but to continue clinical management at the same facility. Women and relatives would try to convince them that they, as health workers, would be able to manage the woman with the complication without referral. One MCH aid stated:

> *"They will want us to do everything while we don't have that ability."* (female SECHN, CHC)

A CHA voiced his frustration at the women's and relatives' behaviour and stated that it endangered his own work.

3) Women's compliance with referral

Influential stakeholders involved to improve compliance. Referral to a district hospital was perceived to come with many fears and worries for a pregnant woman. Examples mentioned by health workers included fear of undergoing surgery, viral haemorrhagic fever (Ebola or Lassa virus) infection, blood donation, male health workers and an unfamiliar environment in terms of language and people. Such fears contributed to women returning home instead of travelling to the health centre they were referred to. Respondents identified three influential stakeholders who may potentially reduce fears around referral. The first stakeholder was the chief of the village or town, whose advice and instruction were of substantial influence on the women's and relatives' referral compliance. The second group of stakeholders, recognised by health workers as similarly influential, consisted of relatives in Freetown, the capital of Sierra Leone. One state-enrolled community health nurse (SECHN) stated:

For peer review only - http://bmjopen.bmj.com/site/about/guidelines.xhtml

"They will always listen to their relatives out there." (female SECHN, MCHP)

These relatives in Freetown were contacted and requested to attempt to convince the woman of the necessity of the referral and to adhere to the referral instructions. Finally, according to the health workers, women who had previously been referred to a district hospital and returned safely had a positive influence on a woman's perceptions regarding referrals to district hospitals.

4) Logistical constraints

Medicine shortage as a burden on the referral system. The logistical constraints of dealing with stockouts of medication in PHUs comprised an additional burden on the referral system. Many health workers, especially those working in CHCs, complained of struggling with medication shortages. Injectable antibiotics were often mentioned as insufficient for the purposed term. When medication stocks had been exhausted, the health worker was faced with two options. The first option was to request money from the woman to purchase medication at a local pharmacy. One health worker described the friction this created with the Free Health Care Scheme for pregnant and lactating women, since it was by law illegal to request money from these women.

"I am not going to ask her to pay for the service I am rendering but just to provide the drug. But me that is punishable crime, I cannot… Then I be in fault." (male CHA, CHC)

However, sending a woman directly to the pharmacy to buy medication themselves was not safe according to several health workers. They expressed their distrust in local pharmacists as they suspected them of not being properly trained and sometimes giving the wrong medication as well as administering injectable drugs themselves against regulations.

For peer review only - http://bmjopen.bmj.com/site/about/guidelines.xhtml

Health workers were therefore often forced to resort to referral to a district hospital in order for a woman to access the correct medication. The CHA voiced his desperation:

> *"So what would you do? You just have to refer."* (male CHA, CHC)

He also expressed his worry about the reaction from the district hospital after receiving such referrals. He feared that the district hospital would doubt the competency of the health workers at the PHU referring a woman who could potentially be managed at their own facility. Finally, some respondents pointed out that referring such a woman exposed them to additional adverse outcomes such as the relatives falling back on traditional medicine or going to a local pharmacy, since this was cheaper than paying for referral transportation.

Inadequate ambulance availability. Tonkolili District has a limited number of ambulances available for the transport of women with emergency obstetric complications. Many health workers complained about the fact that when they call for an ambulance, they are told that the ambulance has broken down or to wait since the ambulance is on its way to a different, sometimes very distant PHU. It was also noted that it sometimes takes a long time before the ambulance team, comprised of a driver and a nurse, is mobilised at the district hospital and the ambulance is finally under way. One CHA summarised the problem as:

> *"So sometimes it's very difficult; the time the ambulance is here, the patient is seriously in a critical condition."* (male CHA, CHC)

Another problem reported was the road accessibility of certain PHUs. Some of these PHUs can only be reached by motorbike and on foot. Accessibility is worse during rainy season. Ambulance transport during the rainy season was even stated to be not possible at all for several PHUs.

For peer review only - http://bmjopen.bmj.com/site/about/guidelines.xhtml

DISCUSSION

This study highlights several aspects of the obstetric referral system in rural Sierra Leone, which require attention in order to provide timely and adequate management of women with emergency obstetric complications.

Importance of communication between staff of different health centres

The importance of communication between health centres to achieve an effective referral system was widely acknowledged by participating providers. Health care workers generally concurred with each other on the advantages of receiving advice on whether to continue management, or to refer a woman instead. However, our findings concerning the practice of waiting for a higher-cadre health worker from a different health facility to arrive and personally examine the woman before advising on a referral decision, are in disagreement with protocol and would increase type 2 delay. This delay can be largely abated by adequate use of mobile phones to communicate with higher-cadre health workers for advice regarding referral decisions [15], as well as further education for MCH aids who will not need a second opinion anymore. Furthermore, the reluctance of lower-cadre health workers in referring women directly to a district hospital and thereby bypassing the higher-cadre health worker's judgment as well as the reluctance of referring women to a CHC with the advice to further refer to a district hospital reveal potentially harmful hierarchy between health workers. From Tanzania, Ueno et al. reported a similar atmosphere of hierarchy and lack of cooperation between different cadres of health workers and levels of health facilities as a challenge to EmOC service delivery [16].

Our findings also imply that following referral, district hospitals need to take initiative in providing health workers in PHUs with feedback in order to improve and encourage future

For peer review only - http://bmjopen.bmj.com/site/about/guidelines.xhtml

referrals and follow-up management of the woman after discharge from the district hospital. Studies in Ghana, Burundi and Northern Uganda reported similar demands for feedback after referral [17-19]. Multiple other studies have described related gaps in communication surrounding obstetric referrals [20, 21] and have specified the critical role of communication in an effective referral system [21-24]. Improvement in communication between health facilities and health workers is a necessary first step towards improving the referral system in northern Tonkolili District.

Underlying influences on decision-making

Our findings point at a mindset of some health workers regarding obstetric referrals that has potential adverse effects on timely management of women with emergency obstetric complications. Maternal mortality and morbidity were seen as tragic events for women and relatives, but also alarming for themselves as health workers. Referral and the ensuing transfer of responsibility was regarded as an option to prevent themselves from being blamed in case of a complication. It is hypothesised that this mind-set is an adverse result of the increased awareness of, and attention to, the high maternal mortality and morbidity rates in Sierra Leone. Obstetric audits have been proven to be an effective method of reducing maternal mortality and morbidity [25-27], but a negative impact on work satisfaction and motivation have also been reported [27, 28]. However, our findings provide limited evidence and further research in rural Sierra Leone is essential to accurately analyse this information.

Another underlying influence which became apparent throughout the interviews was the persuasiveness of women and relatives who did not want to be referred to a different health facility. Such persuasiveness has potential to delay the referral decision made by a health

For peer review only

worker and thus results in phase 1 and phase 2 delays. Also, it may lead to over-confidence of lower-cadre health workers respecting their ability in managing women with obstetric complications, as has been previously reported in Sierra Leone by Theuring et al. [13].

Improving women's compliance with referral

Fears experienced by women, as reported by health workers, to accept referral to a district hospital such as fear of operations, blood donation, male health workers conducting births, and a new environment were largely similar to those previously reported by multiple studies in similar settings [17, 19, 29, 30]. A lingering fear of Ebola in district hospitals in post-Ebola regions including Sierra Leone has been described by several other studies [13, 31, 32]. Three groups of influential stakeholders were identified: local village chiefs, relatives in the capital, Freetown; and women who have previously been referred to a district hospital and have returned safely. Consulting these stakeholders in case of referral refusal will potentially increase women's compliance. These stakeholders are understood to be similarly influential in all rural areas of Sierra Leone.

Logistical constraints

The influence of medication shortage in PHUs on the effectiveness of the referral system becomes apparent through our findings. The combined effect of the Free Health Care Scheme [33] and the distrust in local pharmacies forces health workers into avoidable referrals to district hospitals. In turn, these referrals lead women to a choice of options from traditional medication or buying medication from a local pharmacy. The shortage of medication in PHUs is a burden on and complicating factor of the referral system in northern Tonkolili District; however, adequate availability of medication should not avert mandatory

For peer review only

referrals of women with obstetric complications, which require management in district hospitals.

The shortage of ambulances for transport of women from lower-level health facilities to district hospitals is a commonly reported contributor to phase 2 delay in sub-Saharan Africa [17-19, 34, 35]. Our findings show that this barrier to access to adequate emergency obstetric care is also present in rural Sierra Leone. The inability of ambulances to reach certain PHUs, due to arduous terrain such as steep hills and river crossings and poor road conditions, worsened by seasonal rains, displays the poor infrastructure of rural Sierra Leone.

In February 2019, after our data collection was completed, the Ministry of Health and Sanitation of Sierra Leone and the non-governmental organisation Doctors with Africa CUAMM launched the National Emergency Medical Service (NEMS), which provides free-of-charge ambulance service in all of Sierra Leone. The implementation of the NEMS allows for imperative reduction in delay in access to adequate emergency obstetric care in district hospitals. This reduction in delay is possible as the number of functioning ambulances is increased, and as the travel time is decreased as soon as the ambulances are not only stationed in district hospitals any longer, but also in PHUs.

Strengths and Limitations

A strength of this study is the selection of PHUs and health workers for the interviews, which is representative of health facilities and health workers in rural Sierra Leone. Therefore, interventions targeting the obstetric referral system in other areas of rural Sierra Leone can be supported by our findings. Another strength is the use of open-ended questions during the interviews, which allowed the participants to express their own experiences and feelings.

For peer review only

Additionally, as this study was conducted before the implementation of the NEMS, this study allows for a follow-up study analysing the effect of the NEMS. The authors hypothesise that only the themes 'communication' and 'logistical constraints' have been affected by the NEMS.

A limitation of this study was that the data collected using semi-structured interviews was not triangulated with quantitative data collected with other methods. Additionally, a limited number of interviews were conducted which lowers the quality of evidence. Lastly, the interviews were conducted in English while the participants were more familiar with Krio.

CONCLUSION

In the perspectives of health care workers, delay in access to adequate emergency obstetric care is caused by lack of communication between health workers at different facilities, lack of involvement of influential stakeholders, medication shortage and lack of ambulance services. Of note, fear among women and their relatives for them to be referred is another cause of delay. Furthermore, the decision-making of health care workers concerning referral is negatively influenced by an atmosphere of hierarchy and fear of having maternal deaths and other severe complications at their facility.

Interventions that may reduce delay in access to adequate emergency obstetric care include communication by mobile phones for advice regarding referral decisions and for feedback after a referral decision has been made. Involvement of influential stakeholders to increase women's compliance to referral is an additional intervention that may be considered.

This study highlights factors that may complicate timely and adequate referral of women in need of emergency obstetric care. As this delay is an underlying cause of the high MMR in

For peer review only

For peer review only - http://bmjopen.bmj.com/site/about/guidelines.xhtml

rural Sierra Leone, these potential sources and causes of delay must be considered in efforts to reduce maternal mortality.

LIST OF ABBREVIATIONS

MMR: Maternal Mortality Ratio;

BEmONC: Basic Emergency Obstetric and Neonatal Care;

CEmONC: Comprehensive Emergency Obstetric and Neonatal Care;

MCHP: Maternal and Child Health Post;

CHP: Community Health Post;

CHC: Community Health Centre;

PHU: Peripheral Health Unit;

CHO: Community Health Officer;

CHA: Community Health Assistant;

SECHN: State-Enrolled Community Health Nurse;

MCH-aid: Maternal and Child Health Aid;

NEMS: National Emergency Medical Service

DECLARATIONS

Ethics approval and consent to participate

The study proposal was endorsed by the Masanga Medical Research Unit Scientific Review Committee. Ethical approval was obtained from the Sierra Leone Ethics and Scientific Review Committee. Permission to conduct the study in Tonkolili District was obtained from the

For peer review only - http://bmjopen.bmj.com/site/about/guidelines.xhtml

For peer review only

District Health Management Team. Written informed consent was obtained from interview participants.

Data availability

The datasets used and/or analysed during the current study are available from the corresponding author on reasonable request.

Competing Interests

All authors declare that they have no competing interests.

Funding

This research received no specific grant from any funding agency in the public, commercial or not-for-profit sectors.

Author Contributions

RP carried out the field work, analysed the data and wrote the first draft of the paper. HM and JvN conceived the study and contributed to phrasing the study question, data interpretation and writing of the paper. AF, PSK and MPG contributed to data interpretation and writing of the paper. TvdA oversaw the conduct of the study and contributed to data interpretation and writing of the paper. All authors have contributed to the writing of, and approved the final version of the paper.

Acknowledgements

The authors wish to acknowledge all the staff in health care facilities that participated in the study.

REFERENCES

For peer review only

For peer review only - http://bmjopen.bmj.com/site/about/guidelines.xhtml

1. Statistics Sierra Leone (SSL) and ICF International. Sierra Leone Demographic and Health Survey 2013; Freetown, Sierra Leone and Rockville, Maryland, USA: SSL and ICF International; 2014 [Available from: https://dhsprogram.com/pubs/pdf/fr297/fr297.pdf accessed February 2019.

2. United Nations. Sustainable Development Goals 2015 [Available from: https://www.un.org/sustainabledevelopment/sustainable-development-goals/ accessed July 1 2019.

3. Sierra Leone Ministry of Health and Sanitation. Sierra Leone National Reproductive, Maternal, Newborn, Child and Adolescent Health Strategy 2017-2021 [Available from: https://www.afro.who.int/publications/sierra-leone-national-reproductive-maternal-newborn-child-and-adolescent-health accessed February 2019.

4. Sochas L, Channon AA, Nam S. Counting indirect crisis-related deaths in the context of a low-resilience health system: the case of maternal and neonatal health during the Ebola epidemic in Sierra Leone. *Health Policy and Planning* 2017;32(Suppl 3):32-39. doi: 10.1093/heapol/czx108 [published Online First: 2017/11/18]

5. Huizenga E, van der Ende J, Zwinkels N, et al. A Modified Case Definition to Facilitate Essential Hospital Care During Ebola Outbreaks. *Clin Infect Dis* 2019;68(10):1763-68. doi: 10.1093/cid/ciy798 [published Online First: 2018/09/22]

6. Treacy L, Bolkan HA, Sagbakken M. Distance, accessibility and costs. Decision-making during childbirth in rural Sierra Leone: A qualitative study. *PLoS One* 2018;13(2):e0188280. doi: 10.1371/journal.pone.0188280 [published Online First: 2018/02/21]

7. Thaddeus S, Maine D. Too far to walk: maternal mortality in context. *Soc Sci Med* 1994;38(8):1091-110. [published Online First: 1994/04/01]

For peer review only - http://bmjopen.bmj.com/site/about/guidelines.xhtml

8. Geleto A, Chojenta C, Musa A, et al. Barriers to access and utilization of emergency obstetric care at health facilities in sub-Saharan Africa: a systematic review of literature. *Syst Rev* 2018;7(1):183. doi: 10.1186/s13643-018-0842-2 [published Online First: 2018/11/15]

9. Tong A, Sainsbury P, Craig J. Consolidated criteria for reporting qualitative research (COREQ): a 32-item checklist for interviews and focus groups. *Int J Qual Health Care* 2007;19(6):349-57. doi: 10.1093/intqhc/mzm042 [published Online First: 2007/09/18]

10. Ameh C, Msuya S, Hofman J, et al. Status of emergency obstetric care in six developing countries five years before the MDG targets for maternal and newborn health. *PLoS One* 2012;7(12):e49938. doi: 10.1371/journal.pone.0049938 [published Online First: 2012/12/14]

11. Bailey PE, van Roosmalen J, Mola G, et al. Assisted vaginal delivery in low and middle income countries: an overview. *Bjog* 2017;124(9):1335-44. doi: 10.1111/1471-0528.14477 [published Online First: 2017/02/01]

12. Ministry of Health and Sanitation and UNFPA. Emergency Obstetric and Neonatal Care Guidelines.

13. Theuring S, Koroma AP, Harms G. "In the hospital, there will be nobody to pamper me": a qualitative assessment on barriers to facility-based delivery in post-Ebola Sierra Leone. *Reprod Health* 2018;15(1):155. doi: 10.1186/s12978-018-0601-9 [published Online First: 2018/09/17]

14. Malterud K. Systematic text condensation: a strategy for qualitative analysis. *Scand J Public Health* 2012;40(8):795-805. doi: 10.1177/1403494812465030 [published Online First: 2012/12/12]

For peer review only

15. Noordam AC, Kuepper BM, Stekelenburg J, et al. Improvement of maternal health services through the use of mobile phones. *Trop Med Int Health* 2011;16(5):622-6. doi: 10.1111/j.1365-3156.2011.02747.x [published Online First: 2011/02/24]

16. Ueno E, Adegoke AA, Masenga G, et al. Skilled birth attendants in Tanzania: a descriptive study of cadres and emergency obstetric care signal functions performed. *Matern Child Health J* 2015;19(1):155-69. doi: 10.1007/s10995-014-1506-z [published Online First: 2014/05/06]

17. Nwameme AU, Phillips JF, Adongo PB. Compliance with emergency obstetric care referrals among pregnant women in an urban informal settlement of Accra, Ghana. *Matern Child Health J* 2014;18(6):1403-12. doi: 10.1007/s10995-013-1380-0 [published Online First: 2013/11/02]

18. Chi PC, Bulage P, Urdal H, et al. Barriers in the Delivery of Emergency Obstetric and Neonatal Care in Post-Conflict Africa: Qualitative Case Studies of Burundi and Northern Uganda. *PLoS One* 2015;10(9):e0139120. doi: 10.1371/journal.pone.0139120 [published Online First: 2015/09/26]

19. Afari H, Hirschhorn LR, Michaelis A, et al. Quality improvement in emergency obstetric referrals: qualitative study of provider perspectives in Assin North District, Ghana. *BMJ Open* 2014;4(5):e005052. doi: 10.1136/bmjopen-2014-005052 [published Online First: 2014/05/17]

20. Austin A, Gulema H, Belizan M, et al. Barriers to providing quality emergency obstetric care in Addis Ababa, Ethiopia: Healthcare providers' perspectives on training, referrals and supervision, a mixed methods study. *BMC Pregnancy Childbirth* 2015;15:74. doi: 10.1186/s12884-015-0493-4 [published Online First: 2015/04/18]

For peer review only

514 21. Holmes W, Kennedy E. Reaching emergency obstetric care: overcoming the 'second
515 delay'. Melbourne, Australia: Burnet Institute on behalf of Compass, the Women's
516 and Children's Health Knowledge Hub, 2010.
517 22. Tayler-Smith K, Zachariah R, Manzi M, et al. An ambulance referral network improves
518 access to emergency obstetric and neonatal care in a district of rural Burundi with
519 high maternal mortality. *Trop Med Int Health* 2013;18(8):993-1001. doi:
520 10.1111/tmi.12121 [published Online First: 2013/05/21]
521 23. Tayler-Smith K, Zachariah R, Manzi M, et al. Achieving the millennium development goal
522 of reducing maternal mortality in rural Africa: an experience from Burundi. *Trop Med*
523 *Int Health* 2013;18(2):166-74. doi: 10.1111/tmi.12022 [published Online First:
524 2012/11/21]
525 24. Fournier P, Dumont A, Tourigny C, et al. Improved access to comprehensive emergency
526 obstetric care and its effect on institutional maternal mortality in rural Mali. *Bull*
527 *World Health Organ* 2009;87(1):30-8. [published Online First: 2009/02/07]
528 25. van den Akker T, Mwagomba B, Irlam J, et al. Using audits to reduce the incidence of
529 uterine rupture in a Malawian district hospital. *Int J Gynaecol Obstet*
530 2009;107(3):289-94. doi: 10.1016/j.ijgo.2009.09.005 [published Online First:
531 2009/10/23]
532 26. van den Akker T, van Rhenen J, Mwagomba B, et al. Reduction of severe acute maternal
533 morbidity and maternal mortality in Thyolo District, Malawi: the impact of obstetric
534 audit. *PLoS One* 2011;6(6):e20776. doi: 10.1371/journal.pone.0020776 [published
535 Online First: 2011/06/17]

For peer review only

27. Ivers N, Jamtvedt G, Flottorp S, et al. Audit and feedback: effects on professional practice and healthcare outcomes. *Cochrane Database Syst Rev* 2012(6):Cd000259. doi: 10.1002/14651858.CD000259.pub3 [published Online First: 2012/06/15]

28. Bakker W, van den Akker T, Mwagomba B, et al. Health workers' perceptions of obstetric critical incident audit in Thyolo District, Malawi. *Trop Med Int Health* 2011;16(10):1243-50. doi: 10.1111/j.1365-3156.2011.02832.x [published Online First: 2011/07/20]

29. Wright K, Banke-Thomas A, Sonoiki O, et al. Opinion of women on emergency obstetric care provided in public facilities in Lagos, Nigeria: A qualitative study. *Health Care Women Int* 2017;38(6):527-43. doi: 10.1080/07399332.2016.1234482 [published Online First: 2016/09/10]

30. Liambila WN, Kuria SN. Birth attendance and magnitude of obstetric complications in Western Kenya: a retrospective case-control study. *BMC Pregnancy Childbirth* 2014;14:311. doi: 10.1186/1471-2393-14-311 [published Online First: 2014/09/10]

31. Van Bortel T, Basnayake A, Wurie F, et al. Psychosocial effects of an Ebola outbreak at individual, community and international levels. *Bull World Health Organ* 2016;94(3):210-4. doi: 10.2471/blt.15.158543 [published Online First: 2016/03/12]

32. Jones T, Ho L, Kun KK, et al. Rebuilding people-centred maternal health services in post-Ebola Liberia through participatory action research. *Glob Public Health* 2018;13(11):1650-69. doi: 10.1080/17441692.2018.1427772 [published Online First: 2018/02/01]

33. Wakabi W. Mothers and infants to get free health care in Sierra Leone. *Lancet* 2010;375(9718):882. doi: 10.1016/s0140-6736(10)60371-6 [published Online First: 2010/03/17]

For peer review only

34. Mirkuzie AH, Sisay MM, Reta AT, et al. Current evidence on basic emergency obstetric and newborn care services in Addis Ababa, Ethiopia; a cross sectional study. *BMC Pregnancy Childbirth* 2014;14:354. doi: 10.1186/1471-2393-14-354 [published Online First: 2014/10/11]

35. Echoka E, Makokha A, Dubourg D, et al. Barriers to emergency obstetric care services: accounts of survivors of life threatening obstetric complications in Malindi District, Kenya. *Pan Afr Med J* 2014;17 Suppl 1:4. doi: 10.11694/pamj.supp.2014.17.1.3042 [published Online First: 2014/03/20]

For peer review only

For peer review only - http://bmjopen.bmj.com/site/about/guidelines.xhtml

SUPPLEMENTARY FILE 1

COREQ checklist of reporting

Topic	Item No.	Guide Questions/Description	Reported on Page No.
**Domain 1: Research team and reflexivity**			
Personal characteristics			
Interviewer/facilitator	1	Which author/s conducted the interview or focus group?	8
Credentials	2	What were the researcher's credentials? E.g. PhD, MD	8
Occupation	3	What was their occupation at the time of the study?	8
Gender	4	Was the researcher male or female?	8
Experience and training	5	What experience or training did the researcher have?	8
Relationship with participants			
Relationship established	6	Was a relationship established prior to study commencement?	10
Participant knowledge of the interviewer	7	What did the participants know about the researcher? e.g. personal goals, reasons for doing the research	10
Interviewer characteristics	8	What characteristics were reported about the inter viewer/facilitator? e.g. Bias, assumptions, reasons and interests in the research topic	10
**Domain 2: Study design**			
Theoretical framework			
Methodological orientation and Theory	9	What methodological orientation was stated to underpin the study? e.g. grounded theory, discourse analysis, ethnography, phenomenology, content analysis	11
Participant selection			
Sampling	10	How were participants selected? e.g. purposive, convenience, consecutive, snowball	8, 10
Method of approach	11	How were participants approached? e.g. face-to-face, telephone, mail, email	10
Sample size	12	How many participants were in the study?	10
Non-participation	13	How many people refused to participate or dropped out? Reasons?	10
Setting			
Setting of data collection	14	Where was the data collected? e.g. home, clinic, workplace	6, 9, 11
Presence of non-participants	15	Was anyone else present besides the participants and researchers?	11
Description of sample	16	What are the important characteristics of the sample? e.g. demographic data, date	6, 10
Data collection			
Interview guide	17	Were questions, prompts, guides provided by the authors? Was it pilot tested?	11
Repeat interviews	18	Were repeat inter views carried out? If yes, how many?	11
Audio/visual recording	19	Did the research use audio or visual recording to collect the data?	11
Field notes	20	Were field notes made during and/or after the interview or focus group?	N/A
Duration	21	What was the duration of the inter views or focus group?	11
Data saturation	22	Was data saturation discussed?	N/A
Transcripts returned	23	Were transcripts returned to participants for comment and/or correction?	11

For peer review only

Topic	Item No.	Guide Questions/Description	Reported on Page No.
**Domain 3: analysis and findings**			
Data analysis			
Number of data coders	24	How many data coders coded the data?	11
Description of the coding tree	25	Did authors provide a description of the coding tree?	11
Derivation of themes	26	Were themes identified in advance or derived from the data?	11
Software	27	What software, if applicable, was used to manage the data?	11
Participant checking	28	Did participants provide feedback on the findings?	11
Reporting			
Quotations presented	29	Were participant quotations presented to illustrate the themes/findings? Was each quotation identified? e.g. participant number	12-17
Data and findings consistent	30	Was there consistency between the data presented and the findings?	22
Clarity of major themes	31	Were major themes clearly presented in the findings?	12
Clarity of minor themes	32	Is there a description of diverse cases or discussion of minor themes?	N/A

Developed from: Tong A, Sainsbury P, Craig J. Consolidated criteria for reporting qualitative research (COREQ): a 32-item checklist for interviews and focus groups. *International Journal for Quality in Health Care*. 2007. Volume 19, Number 6: pp. 349 – 357

For peer review only

SUPPLEMENTARY FILE 2

Signal functions of BEmONC and CEmONC

Basic Emergency Obstetric and Neonatal Care (BEmONC)

1. Parenteral treatment of infections (antibiotics)
2. Parenteral treatment of severe pre-eclampsia/eclampsia (e.g., MgSO4)
3. Treatment of PPH (e.g., uterotonics)
4. Manual vacuum aspiration of retained products of conception
5. Assisted vaginal delivery (e.g., vacuum-assisted delivery)
6. Manual removal of placenta
7. New-born resuscitation

Comprehensive Emergency Obstetric and Neonatal Care (CEmONC)

All components of BEmONC, plus

1. Surgical capability, including anaesthesia (e.g., Caesarean Section)
2. Blood transfusion

For peer review only

SUPPLEMENTARY FILE 3

Interview Guide Obstetric Referral System Northern Tonkolili District

General

1. Describe the catchment area of this health facility (number of villages, terrain).
2. Describe this health facility (number and competency of staff, average number of births and referrals).
3. Describe the referral options of this health facility (nearest health facility, nearest CHC, nearest district hospital).

Authorisation

4. Which of the staff is allowed to decide to refer women to another facility?
5. Is there always staff present that is allowed to independently refer women?
6. Does the CHO or midwife in the CHC have to be contacted before referring a woman to another facility?

Referral Process

7. Describe the steps taken when referring a pregnant or post-partum women.
8. Describe how/if referral notes are used in this health facility.
9. Describe the benefit of referral notes.
10. Describe how/if feedback is received from the referral facility.
11. Describe how/if advice is obtained from other health workers before referral.
12. Describe how/if national guidelines are used for referring a woman.
13. Describe how/if maternity patients without an emergency indication are referred to a different health facility.

Accessibility

14. Describe the accessibility of Magburaka Government Hospital from here.
15. Describe the accessibility of Masanga Hospital from here.
16. If you were to refer 10 women to a hospital, how many of them do you think will actually arrive at the hospital?
17. Describe some reasons why referred women sometimes refuse referral.
18. Describe transportation options available for referral.

Ambulance Referrals

19. Describe the process of ordering an ambulance from Magburaka Government Hospital.
20. Describe the process of ordering an ambulance from Masanga Hospital.
21. How many times have you ordered an ambulance in the past month?
22. How long does it take for the ambulance to arrive at your health facility after you have ordered the ambulance? (from Magburaka/Masanga)
23. Which of the staff is allowed to order an ambulance?
24. Is there always staff present that is allowed to order an ambulance?

For peer review only

25. Does the CHO or midwife in the CHC have to be contacted before ordering an ambulance?
26. Is transferral by ambulance free for the woman?
27. Describe problems with the current ambulance referral system.
28. Describe recommendations for improving the ambulance referral system.

ANC/under 5

29. Describe the antenatal care in this health facility (frequency of ANC-days, number of women)
30. Describe how/if governmental Maternity Record Cards are used during ANC visits, delivery and postnatal checks.

Masanga Hospital

31. Describe recommendations for improving the health care at Masanga Hospital.
32. Is Masanga Hospital an official referral hospital in Tonkolili District?
33. Is healthcare free for pregnant and lactating women and children under 5 at Masanga Hospital?

For peer review only

SUPPLEMENTARY FILE 4

Original study protocol

Includes protocol regarding quantitative research which is not included in submitted article

For peer review only

For peer review only - http://bmjopen.bmj.com/site/about/guidelines.xhtml

The current situation of maternal health in Tonkolili District and the perceptions of health workers at peripheral health units in the catchment area of Masanga Hospital concerning the current referral system of obstetric patients

For peer review only

MASANGA RESEARCH

Lead applicant

Ryan Proos, medical student Leiden University Medical Center, Masanga Hospital Sierra Leone

Correspondence address: e-mail: ryanproos9@gmail.com Tel: +232 76557283

Supervisor

Dr. T. van den Akker, Leiden University Medical Center, Netherlands (Gynaecology and Obstetrics)

Research team

Hanna Mathéron, MD Global Health and Tropical Medicine, Medical Officer Masanga Hospital

Teneh Kamara, midwife at Masanga Hospital Sierra Leone

Background

Sierra Leone is one of the most dangerous countries for women to be pregnant and to give birth. According to the Sierra Leone Demographic Health Survey (2013)[1], Sierra Leone has a maternal mortality ratio (MMR) of 1,165 per 100,000 live births, the highest in the world and more than six times the global average. The UN Sustainable Development Goals (SDGs), adopted in 2015, aim for a reduction of the global maternal mortality ratio to less than 70 per 100,000 live births in 2030. In line with these SDGs, the Ministry of Health and Sanitation of Sierra Leone launched the Reproductive, Maternal, Newborn, Child and Adolescent Health (RMNCAH) Strategy in 2017, which aims for a reduction of preventable deaths of women, children and adolescents and ensuring their well-being[2]. The target is to reduce the MMR of 1,165 per 100.000 live births to 650 per 100.000 live births by 2021. With launching this strategy, the government acknowledges the need for change, but there is still a long way to go to achieve this goal.

Masanga Hospital is situated in Tonkolili district, centrally located in the Northern Province of Sierra Leone. The catchment area of Masanga Hospital comprises of approximately 150,000 inhabitants and 15 peripheral health units (PHUs) . Besides Masanga Hospital there are two other hospitals in Tonkolili district, Magburaka Hospital and Lion Heart Hospital in Yele.

Currently, there is no complete and trustworthy overview of maternal health in Tonkolili District. Therefore, a baseline assessment using the WHO Reproductive Health Indicators and the RMNCAH coverage targets is needed to work towards achieving the goal set by the Ministry.

It is hypothesized that the poor referral system is a major causative factor of the current poor maternal health situation in Tonkolili district. Perceptions of health workers at PHUs concerning the referral system of obstetric patients is necessary to identify areas of improvement.

Aims and Objectives

The aim of this research project is to do a retrospective, quantitative descriptive analysis of data collected during project to describe maternal health demography, geography, delivery and complication rates of Tonkolili district, especially in relationship to the catchment area of Masanga Hospital. Additionally, an assessment of health workers' perceptions of the current referral system of obstetric patients will be undertaken.

For peer review only

Research Questions

1. What is the current maternal health situation in Tonkolili District, Sierra Leone, in terms of WHO Reproductive Health Indicators and the RMNCAH coverage target matches?

The baseline assessment shall provide an overview of several WHO Reproductive Health Indicators and RMNCAH coverage targets in Tonkolili district over the period 2016-2018**.** Data collection will be done in the Peripheral Health Units (PHUs) and the district hospitals (Masanga Hospital, Magburaka Hospital and Lion Heart Hospital, Yele), in collaboration with the District Health Sister (DHS1), District Medical Officer (DMO) and the District Health Information System (DHIS).

The following data will be collected per health facility:

- Level of service provision (MCHP, CHP, CHC, District Hospital)
- Location in the catchment area
- Number of deliveries (spontaneous, assisted, CS)
- Number of live births
- Number of still births
- Number of maternal deaths
- Number of complications (antepartum haemorrhage (APH), postpartum haemorrhage (PPH), pregnancy-induced hypertension (PIH), pre-eclampsia, eclampsia, obstructed labour, post-partum sepsis)
- Number of referrals, reasons for referral
- Number of antenatal care visits
- Number of intermittent preventive treatment (IPT) during ANC

2. What are the perceptions of health workers at peripheral health units in the catchment area of Masanga Hospital concerning the current referral system of obstetric patients?

Perceptions of health workers working in PHUs in the catchment area of Masanga Hospital will be explored in several discussions.

The discussions will focus on the following topics:

- Communication between health centres
- Determinants of referral decision
- Actions taken before and during referral
- Effect of antenatal care on referrals
- Referral constraints

For peer review only - http://bmjopen.bmj.com/site/about/guidelines.xhtml

Study Design

This research project is a retrospective, quantitative descriptive analysis. Data collection will be done in collaboration with the District Health Sister (DHS1), District Medical Officer (DMO) and the DHIS.

Patients/Participants

All women that visited a PHU or hospital in Tonkolili district for antenatal care visits, health facility deliveries or pregnancy or delivery complications during the period 2016-2018 will be included in the baseline overview. Selection bias must be considered as not all pregnant women visit a health facility. Reproductive health data is available through the centralized District Health Information System. Access to the District Health Information System will be officially requested. Data is available of approximately 12,000 facility-based live births in the district per year and therefore roughly 36,000 facility-based live births in the specified period. However, it is estimated that 46% of pregnant women in Sierra Leone give birth at home[2]. There is therefore no data available of these homebirths, but these women might have visited a health facility for antenatal care or for post-partum complications.

Health workers at PHUs in the catchment area of Masanga Hospital will be asked to participate in the group discussions focusing on their perceptions of the current obstetric referral system. Written informed consent will be obtained using the attached form. An interview guide will be used.

Statistical Methods

After centralization of the data in SPSS, a baseline overview of the variables named for research question 1 will be made for each PHU and hospital and for Tonkolili district. The qualitative data of the group discussions will be analyzed using systematic text condensation.

For peer review only - http://bmjopen.bmj.com/site/about/guidelines.xhtml

Results

The baseline assessment shall provide an overview of several WHO Reproductive Health Indicators and the RMNCAH coverage targets in Tonkolili district over the period 2016-2018. Regional differences in maternal health outcomes in Tonkolili District will be identified. These regional differences will be linked to the accessibility of emergency obstetric care in those regions. This baseline overview will provide a basis for further research and implementation of new interventions, aimed at reaching the goal set by the Ministry.

The perceptions of health workers at PHUs in the catchment area of Masanga Hospital will illuminate constraints of the current referral system of obstetric patients. Additionally, the quality of communication between different levels of health facilities concerning referrals will be described. Possible solutions as suggested by the health workers will be described.

The results will be summarized in a descriptive article.

For peer review only

Ethical Considerations

Ethical Approval

Ethical approval will be obtained from the Sierra Leone Ethics and Scientific Review Committee.

Selection of study population and recruitment of research participants

All patients registered at PHUs and hospitals in Tonkolili District for antenatal care visits, deliveries, and pregnancy complications in the period 2016-2018 are selected and included. Patients are free to refuse participation. This refutation must be apparent from the District Health Information System.

Informed consent process

Receiving medical care at a PHU or hospital implicitly indicates given consent to use of registered data for medical research through the District Health Information System. Written informed consent will be obtained from participants in the group discussions. The informed consent form is included in this application.

Risks of participation

Participation in the baseline overview exposes patients to minimal chance of harm. Participation does not include any clinical intervention. Confidentiality and anonymity of patient data is maximally safeguarded.

Participation in the group discussions exposes the health workers to minimal chance of harm. Confidentiality and anonymity of participant data is maximally safeguarded.

Inducements, financial benefits, and financial costs

Participation is not rewarded with any financial, material or healthcare service benefit.

Protection of research participants' privacy and confidentiality

Research participant data is centrally stored in the District Health Information System. This digital system is password protected. To ensure confidentiality, this data is only accessible by the head researcher and supervisors.

Group discussion participant data is stored on password-protected computers in Masanga Hospital. Only the head researcher has access to the names, functions and health facilities of the participants. Reported data is anonymous and cannot be traced back to the specific health facility.

Independence of research and conflicts of interests

Research is conducted on behalf of Masanga Hospital and in collaboration with Tonkolili District. No other parties are involved. The researchers certify that they have no conflicts of interests.

For peer review only - http://bmjopen.bmj.com/site/about/guidelines.xhtml

Schedule

1-8 weeks:

- Contact with DMO and DHS, attending the district meetings and the district maternal mortality meetings in Magburaka
- Application for funding
- Application for District Health Information System access

8-20 weeks

- Analysation of DHIS data
- Group discussions

20-28 weeks

- Analysation and illustration of DHIS data
- Writing descriptive article

GANTT chart

	W1-4	W5-8	W9-12	W13-16	W17-20	W20-24	W24-28
**DMO/DHS contact**	■						
**Initiation visits in PHUs/Hospitals**	■	■					
**DHIS access application**	■	■					
**Group discussions**			■	■	■		
**Data analysis**			■	■	■	■	■
**Writing**					■	■	■
**Submitting article**							■

For peer review only

For peer review only - http://bmjopen.bmj.com/site/about/guidelines.xhtml

References

1. Sierra Leone Demographic and Health Survey 2013 via the DHS Program STATcompiler. (http://www.statcompiler.com).
2. Sierra Leone Reproductive, Maternal, Newborn, Child and Adolescent Health (RMNCAH) strategy 2017-2021, Ministry of Health Sierra Leone

For peer review only

For peer review only - http://bmjopen.bmj.com/site/about/guidelines.xhtml

MASANGA RESEARCH

Participant Informed Consent Form

Study title: Perceptions of health workers at peripheral health units in the catchment area of Masanga Hospital concerning the current referral system of obstetric patients

Researcher: Ryan Proos E-mail: ryanproos9@gmail.com

Institute: Masanga Medical Research Unit, Sierra Leone

	Add your initials if you agree
I agree that this interview will be recorded and that the data will be analyzed and reported anonymously.	
I understand that I can withdraw from this study at any time, without given reason and all my data will be deleted.	
I understand I can contact the Sierra Leone Ethics and Scientific Review Committee at any time (contact details below*).	

Name of participant: ……………………………………………………………………………….

Signature: …………………………………………… Date: ……………………………….

Name of person taking consent: ……………………………………………………………….

Signature: …………………………………………… Date: ……………………………….

* Contact details Sierra Leone Ethics and Scientific Review Committee:

E-mail: efoday@health.gov.sl

** this form needs to be signed and dated in presence of the patient. If preferred, the patient will receive a copy of this form. The forms will be stored in a secure location, ensuring patients privacy.

For peer review only - http://bmjopen.bmj.com/site/about/guidelines.xhtml

BMJ Open

Perspectives of health workers on the referral of women with obstetric complications: a qualitative study in rural Sierra Leone

Journal:	*BMJ Open*
Manuscript ID	bmjopen-2020-041746.R1
Article Type:	Original research
Date Submitted by the Author:	02-Oct-2020

Complete List of Authors:	Proos, Ryan; Masanga Medical Research Unit; Leiden University Medical Center, Obstetrics and Gynaecology Department<br>Matheron, Hanna; Masanga Medical Research Unit<br>Vas Nunes, Jonathan; Masanga Medical Research Unit<br>Falama, Abdul; Tonkolili District Health Management Team<br>Sery Kamal, Patricia; Tonkolili District Health Management Team<br>Grobusch, Martin; Amsterdam University Medical Centres, Center of Tropical Medicine and Travel Medicine, Department of Infectious Diseases; Masanga Medical Research Unit<br>van den Akker, Thomas; Leiden University Medical Center, Obstetrics and Gynaecology Department; VU Amsterdam, Athena Institute

**Primary Subject Heading**:	Health services research
Secondary Subject Heading:	Health services research, Obstetrics and gynaecology, Public health, Qualitative research, Global health
Keywords:	Maternal medicine < OBSTETRICS, PUBLIC HEALTH, Organisation of health services < HEALTH SERVICES ADMINISTRATION & MANAGEMENT, QUALITATIVE RESEARCH

SCHOLARONE™
Manuscripts

I, the Submitting Author has the right to grant and does grant on behalf of all authors of the Work (as defined in the below author licence), an exclusive licence and/or a non-exclusive licence for contributions from authors who are: i) UK Crown employees; ii) where BMJ has agreed a CC-BY licence shall apply, and/or iii) in accordance with the terms applicable for US Federal Government officers or employees acting as part of their official duties; on a worldwide, perpetual, irrevocable, royalty-free basis to BMJ Publishing Group Ltd ("BMJ") its licensees and where the relevant Journal is co-owned by BMJ to the co-owners of the Journal, to publish the Work in this journal and any other BMJ products and to exploit all rights, as set out in our licence.

The Submitting Author accepts and understands that any supply made under these terms is made by BMJ to the Submitting Author unless you are acting as an employee on behalf of your employer or a postgraduate student of an affiliated institution which is paying any applicable article publishing charge ("APC") for Open Access articles. Where the Submitting Author wishes to make the Work available on an Open Access basis (and intends to pay the relevant APC), the terms of reuse of such Open Access shall be governed by a Creative Commons licence – details of these licences and which Creative Commons licence will apply to this Work are set out in our licence referred to above.

Other than as permitted in any relevant BMJ Author's Self Archiving Policies, I confirm this Work has not been accepted for publication elsewhere, is not being considered for publication elsewhere and does not duplicate material already published. I confirm all authors consent to publication of this Work and authorise the granting of this licence.

For peer review only

Perspectives of health workers on the referral of women with obstetric complications: a qualitative study in rural Sierra Leone

Ryan Proos[1,3], Hanna Mathéron[1,2,4], Jonathan Vas Nunes[1,2,4], Abdul Falama[5], Patricia Sery Kamal[5], Martin Peter Grobusch[1,4*], Thomas van den Akker[3,6]

[1]Masanga Medical Research Unit, Masanga, Sierra Leone

[2]Masanga Hospital, Masanga, Sierra Leone

[3]Obstetrics and Gynaecology Department, Leiden University Medical Center, Leiden, The Netherlands

[4]Center for Tropical Medicine and Travel Medicine, Department of Infectious Diseases, Amsterdam University Medical Centers location AMC, Amsterdam Public Health, Amsterdam Infection & Immunity, Amsterdam, The Netherlands

[5]Tonkolili District Health Management Team, Magburaka, Sierra Leone

[6]Athena Institute, VU University, Amsterdam, The Netherlands

* Corresponding author: Prof. Dr. Martin Peter Grobusch, AUMC location AMC, Meibergdreef 9, 1105 AZ Amsterdam, The Netherlands; email: m.p.grobusch@amsterdamumc.nl

Word count: Abstract – 294 words; Main text body - 4,528 words

For peer review only

ABSTRACT

Objectives

Sierra Leone has one of the highest maternal mortality ratios in the world. Timely and well-coordinated referrals are necessary to reduce delays in providing adequate care for women with obstetric complications. This study describes factors affecting timely and adequate referral of women with obstetric complications in rural areas of Sierra Leone as viewed by health workers in rural health facilities.

Design

A qualitative research with semi-structured interviews using open-ended questions. Data were analysed by systematic text condensation.

Setting

Interviews were held in nine peripheral health units in rural Sierra Leone.

Participants

19 health workers including nurses, midwives and clinical health officers participated in nine interviews.

Results

From the interviews, four major themes describing possible factors of delay in referral of women in need of emergency obstetric care emerged: 1) communication between health care workers; 2) underlying influences on decision-making; 3) women's compliance to referral; 4) logistic constraints.

For peer review only

Several factors in rural Sierra Leone are perceived to complicate timely and adequate referral of women in need of emergency obstetric care. Notable among these factors are fear among women for being referred and fear among health care workers for having maternal deaths or severe obstetric complications occurring at their own facilities. Furthermore, decision-making of health care workers whether to refer a woman or not is negatively influenced by a hierarchical culture with high power distance between health care workers.

Conclusion

Factors identified that complicate timely and adequate referral of women in need of emergency obstetric care must be considered in efforts to reduce maternal mortality. Possible interventions that may reduce delay in referral include increased communication by mobile phones between health workers for advice and feedback regarding referrals, involvement of influential stakeholders to increase women's compliance to referral, and consistent use of standardised management protocols.

For peer review only

ARTICLE SUMMARY

Strengths and limitations of this study

- A strength of this study is the purposive and homogenous sampling used for the selection of peripheral health units and health workers for the interviews, which is representative of health facilities and health workers in rural Sierra Leone.

- A strength of this study is the use of open-ended questions alongside an interview guide, ensuring specific topics were discussed while allowing participants to introduce and discuss additional topics.
- A limitation of this study is that the data collected using semi-structured interviews with health workers were not triangulated with data from interviews with women and relatives.

KEYWORDS

health worker; maternal health; obstetric complications; qualitative study; referral; Sierra Leone

For peer review only

For peer review only - http://bmjopen.bmj.com/site/about/guidelines.xhtml

BACKGROUND

Sierra Leone has one of the highest maternal mortality ratios (MMR) in the world. According to the Sierra Leone Demographic Health survey in 2013, the MMR was 1,165 per 100,000 live births [1]. The need to reduce this extremely high ratio is evident. In order to work towards meeting the United Nations Sustainable Development Goal target of an MMR below 70 per 100 000 [2], the Ministry of Health and Sanitation of Sierra Leone launched the Reproductive, Maternal, Newborn, Child and Adolescent Health Strategy in 2017, aiming to reduce the MMR of 1,165 to 650 by 2021 [3].

An underlying factor of the high MMR in Sierra Leone is the persistent low rate of facility-based births [1]. Country-wide in 2013, only slightly more than half of the women gave birth in a health facility; 49.7% of women in rural areas versus 68.1% in urban areas [1]. The West-African Ebola outbreak from 2013 to 2016 led to a further reduction in facility-based births as a consequence of reduced possibilities and fear to access the health system during the crisis [4, 5].

Pregnant women in Sierra Leone face many barriers to facility-based birth, including long distances, inability to afford costs of transport and healthcare and lack of trust in health facilities [6]. These factors contribute to a phase-1 delay in deciding to seek healthcare in case of an emergency obstetric complication, as described in the three-phase delay model by Thaddeus and Maine [7]. Even after having decided to seek health care, women are often confronted with delays in phase 2 (transport delay), and phase 3 (delay in diagnosis and treatment at the facility) [8]. Shortly before this study was conducted, a new ambulance system was implemented in Sierra Leone, possibly reducing transport delays. Timely and well-coordinated referrals are necessary to further reduce delay in receiving adequate

For peer review only

obstetric care for women with obstetric complications. Currently, there is no literature available analysing aspects of reasons for delay of care within the obstetric referral system in rural Sierra Leone.

This study describes factors affecting timely and adequate referral of women with obstetric complications in rural areas of Sierra Leone through the perspectives of health workers in rural health facilities. This data will provide better understanding of challenges within the health system faced by women who are referred with emergency obstetric complications in rural Sierra Leone.

METHODS

Study design and setting

This qualitative study using semi-structured interviews and following the Consolidated criteria for reporting qualitative research (COREQ) checklist [9] (Supplementary File 1) was conducted between the 1st of September 2018 and the 15th of March 2019 in Tonkolili District. This district is located in the Northern Province of Sierra Leone and sub-divided into eleven chiefdoms. Three hospitals with Comprehensive Emergency Obstetric and Neonatal Care are located in Tonkolili District. Basic and Comprehensive Emergency Obstetric and Neonatal Care (BEmONC and CEmONC) are services fundamental to provide adequate health care during pregnancy and childbirth. The signal functions of BEmONC and CEmONC centres are summarised in Supplementary File 2. Three chiefdoms in the north of Tonkolili District, Kafe Simiria, Kalansogoia and Sambaya Bendugu, with a combined population of 113,521 (2018), are served by two CEmONC centres, Magburaka Government Hospital and Masanga Hospital, an NGO-supported government hospital. Besides the two CEmONC centres, these chiefdoms are served by fifteen peripheral health units (PHUs), including four BEmONC

For peer review only - http://bmjopen.bmj.com/site/about/guidelines.xhtml

centres. The maternity services provided at each facility level in these chiefdoms, according to the Ministry of Health and Sanitation, are summarised in Table 1.

Table 1: Maternity services purportedly provided at each level of health facility

Maternal and Child Health Post (MCHP)	• Antenatal care<br>○ Nutritional supplementation in pregnancy (e.g. iron, folic acid and multivitamins)<br>○ Risk selection and ensuing referral<br>○ Malaria intermittent preventive treatment<br>• Intra- and postpartum care<br>○ Monitoring of labour by using the partograph<br>○ Cord clamping<br>○ Active management of the third stage of labour<br>• Postnatal care<br>○ Clinical assessment of the neonate (e.g. fever, convulsions, feeding)<br>○ Exclusive breastfeeding recommendation<br>○ Cord care<br>○ Clinical assessment of mother (e.g. temperature, blood pressure, bleeding)<br>○ Family planning counselling
Community Health Post (CHP)	• MCHP services (see above)

For peer review only

Community Health Centre (CHC)	• MCHP services (see above)<br>*plus*<br>• Maternal anaemia and urine sediment assessment<br>• BEmONC services (see supplementary file 2)
District Hospital	• MCHP services (see above)<br>*plus*<br>• Maternal anaemia, urine, HIV, malaria and tuberculosis assessment<br>• Ultrasound scan<br>• CEmONC services (see supplementary file 2)

However, not all facilities designated to provide BEmONC and CEmONC in rural Sierra Leone are able to provide the full range of signal functions [10, 11]. Therefore, several BEmONC and CEmONC signal functions might not actually be provided in practice. Referral occurs both in consecutive order starting from Maternal and Child Health Posts (MCHPs) as well as between lower level health facilities and district hospitals. Sierra Leone has national protocols for emergency obstetric care, including referral indications [12].

Tonkolili District was conveniently selected, since the district is located in rural Sierra Leone and the catchment area of the hospital where three of the authors are employed. The chiefdoms in northern Tonkolili District were selected, since these three combined comprise the catchment area for emergency obstetric complications belonging to Masanga Hospital, for reasons of geography such as impassable rivers and mountains and accessibility by road.

The qualitative data used in this study was collected using nine semi-structured interviews conducted by RP (Master of Medicine student, male, first author) between November 2018

For peer review only

131 and January 2019. Purposive sampling was used to ensure variation between selected facilities where interviews were conducted. Selection criteria were that facilities were located in different chiefdoms, provided different levels of care, and had varying accessibilities of the nearest district hospital (Table 2).

Table 2: Facilities where interviews were conducted

Town	Level	Distance to district hospital (km)	Travel time to district hospital* (min)
Chiefdom Kafe Simiria			
Mabontor	CHC	18.9	40
Masumbrie	CHC	21.5	40
Makontande	MCHP	28.9	50
Chiefdom Kalansogoia			
Bumbuna	CHC	42.6	70
Kamasaypana	MCHP	50.0	100
Kemedugu	MCHP	58.5	110
Chiefdom Sambaya Bendugu			
Bendugu	CHC	81.4	150
Kunya	CHP	92.3	180
Dankawalia	MCHP	71.9	135

* Travel time by motorbike during dry season (November – May). Travel time during rainy season (June – October) will be

For peer review only

substantially longer. Travel time by ambulance will be shorter. Roads were unpaved.

Selected facilities were approached either by telephone calls to the in-charge health worker or by face-to-face visits to that health facility. All health workers working at the selected facilities at the time of the interview were invited to participate in the interview. Thus, homogenous sampling was used as each interview was conducted with health workers of different cadres currently working in the same health centre. The number of participants per interview ranged from one to four. In total, nineteen health workers participated in the nine interviews. All participants were explained the relevance and goals of the research. No facility or individual health worker refused participation. Respective health worker cadres and competencies of the participants are summarised in Table 3.

Table 3: Cadres and competencies of respondents

Health worker (number interviewed)	Competencies
Maternal and Child Health aid (MCH aid) (10)	2 years training. Competent in basic obstetric care.
State Enrolled Clinical Health Nurse (SECHN) (2)	2,5 years training. Competent in basic obstetric care.
Community Health Assistant (CHA) (3)	2 years theoretical + 1-year practical training. Competent in basic obstetric care.
Community Health Officer (CHO) (2)	3 years theoretical + 1-year practical training. Competent in basic obstetric care. No training in emergency obstetric care.

For peer review only

Midwife (2)	SECHN training + 1,5 year-midwifery training. Competent in emergency obstetric care including oxytocin administration, manual placenta removal, newborn resuscitation, first treatment for (pre)eclampsia and antibiotic administration. No training in remaining signal functions.

The interview guide (Supplementary File 3) was used as a framework of themes to be discussed during the interviews. The guide was initially developed using themes described by Thaddeus and Maine [7], previous literature concerning pregnancy and childbirth in Sierra Leone [6, 13], and preliminary discussions with stakeholders such as medical officers, community health officers (CHO), midwives and logistical officers employed at district referral hospitals. The guide was piloted in one PHU with two health workers. The interviews were held inside the respective health facilities where only participants and interviewer were present. The interviews were conducted in English, using open-ended questions, and lasted between 30 and 60 minutes each. Data was collected using audio recording. Repeat interviews were not carried out and transcripts were not returned to participants for comments and correction for logistical reasons: there was no funding or practical possibility to re-visit these widely spread-out facilities. The original study protocol (Supplementary File 4) was followed throughout the study.

Study Subject and Public involvement

The District Health Management Team was involved in the design, conduct, reporting and dissemination planning of our research. All participants of the interviews were informed on relevance and goals of the study.

For peer review only

Analysis

All audio recordings of interviews were transcribed verbatim by RP using Express Scribe Transcription Software (NCH Software, Greenwood Village, Colorado, USA). Content analysis was performed by RP through systematic text condensation as described by Malterud [14]. After all interviews were transcribed verbatim, all transcripts were read multiple times to establish an overview of the data. Preliminary themes were identified based on this overview. Hereafter, all transcripts were once again reviewed line by line, to identify 'meaning units', i.e. text fragments containing some information about the research question. These meaning units were marked with a code: a label that connects related meaning units into a code group. These code groups were elaborated from the themes from the first step of the analysis. Hereafter, the meaning units in each code group were connected to form a condensate. Lastly, these condensates were synthesized to accurately reflect the original quotes and some original quotes were included in the text to further illustrate the data. Data analysis was performed manually. HM was involved in the whole process of data analysis and gave feedback on the identification of the preliminary themes, the systematic coding and categorisation of quotes, and the writing of condensates of every theme based on the quotes.

Ethical Considerations

The study proposal was endorsed by the Masanga Medical Research Unit Scientific Review Committee. Ethical approval was obtained from the Sierra Leone National Ethics and Scientific Review Committee on 29 January 2019. Permission to conduct the study in Tonkolili District was obtained from the District Health Management Team. Written informed consent was obtained from interview participants.

For peer review only

RESULTS

From these interviews, four major themes describing possible factors of delay in referral of women in need of emergency obstetric care emerged: 1) communication between health care workers, 2) underlying influences on decision making, 3) women's compliance to referral, 4) logistic constraints.

1) Importance of communication between staff of different health centres

Giving and receiving advice surrounding referrals. Most health workers mentioned the necessity of asking for advice when having to decide whether to continue management or refer the woman to a higher-level health centre. One community health assistant (CHA) explains:

> *"I am not saying 100% I know what I am doing. I know myself. I learn, I can just know my own area and then there are people who know better. I am just a community health officer, assistant in fact."* (male CHA, MCHP)

Advice was often asked for and given in mobile phone conversations. However, sometimes higher cadre health workers travelled to health facilities to review women themselves before advice was given.

> *"Sometimes he will come and he will review the patient and tell us to send the patient, so we call the ambulance."* (female MCH aid, CHP)

Those asked for advice include CHOs, midwives, the District Health Sisters (supervising midwives, members of the Tonkolili District Health Management Team), the head of a maternity ward or a medical officer at a district hospital. However, one maternal and child health aid (MCH aid) mentioned that if she recognized a woman requiring urgent referral to

For peer review only - http://bmjopen.bmj.com/site/about/guidelines.xhtml

a higher-level facility, she would not delay by first calling for advice but rather directly refer the woman to the community health centre (CHC). She would not, however, directly refer the woman to a district hospital or inform the CHC that this woman likely needed onward referral, since she believed that this decision had to be made by CHC staff.

Besides needing advice on whether or not to refer the woman to a higher-level facility, participants also mentioned a need for advice over the phone regarding clinical management while waiting for the ambulance to arrive, since this could take up to several hours.

Feedback after referral. Many health workers indicated that they were interested in the clinical course after a woman had been referred to the district hospital, as illustrated by one CHO:

> *"We are highly interested in feedback, because they are lives and when we call on you people to rescue, then we have interest over them."* (male CHA, CHC)

Health workers in lower level facilities expressed a specific interest in knowing the clinical management including decisions on mode of birth at the district hospital. These health workers were often approached by relatives of the referred woman requesting updates on her clinical condition and outcome. Health workers regularly marked their phone numbers on the referral notes in order to receive feedback. However, many respondents complained never receiving a response from district hospital staff. Instead, they felt forced to call the district hospital themselves, and indicated this comprised a communication barrier, since it required them to spend their own mobile phone credits.

> *"But they don't give us the feedback for us to know if it was a vacuum delivery or what. I have no response..."* (female MCH aid, MCHP)

For peer review only - http://bmjopen.bmj.com/site/about/guidelines.xhtml

Similarly, health workers complained of never receiving discharge notes with follow-up information after the woman had been discharged from the district hospital. They generally relied on the information the woman could give them verbally.

2) Underlying influences on decision-making

Referral, perceived as the safest option for health workers. The necessity of referral, as expressed by one health worker, was often to avoid complications and maternal death occurring at their own facility.

> *"If a maternal death is here, we are going to suffer."* (female SECHN, MCHP)

Often, a referred woman was described as 'not my case'. Another health worker mentioned that when a woman was referred to a CHC, it was up to that facility to manage the woman with the complication and decide what to do. Such transfer of responsibility after referral was further illustrated by a story recounted by an MCH aid about a woman she had recently referred:

> *"Yes, I delivered her. Male baby. Fresh still birth. So it is not my problem, because I have already referred her."* (female MCH aid, MCHP)

Endangering behaviour by women requiring referral. Some women did not want to be referred and health workers were under a lot of pressure from women and relatives while making a referral decision. Women and relatives were at times perceived not to tell the truth when questioned about history, since they wanted to prevent referral. One health worker mentioned that he sometimes heard rumours in the community that the point in time a woman and her relatives indicated as when onset of symptoms occurred was not always correct.

For peer review only

"Nothing of the time stated was really factual, it was not the time, the actual time." (male CHA, MCHP)

Most respondents described that when a woman and her relatives were told that she needed to be referred, they started begging health workers not to refer her but to continue clinical management at the same facility. Women and relatives would try to convince them that they, as health workers, would be able to manage the woman with the complication without referral. One SECHN stated:

"They will want us to do everything while we don't have that ability." (female SECHN, CHC)

A CHA voiced his frustration at the women's and relatives' behaviour and stated that it endangered his own work.

3) Women's compliance with referral

Influential stakeholders involved to improve compliance. Referral to a district hospital was perceived to come with many fears and worries for a pregnant woman. Examples mentioned by health workers included fear of undergoing surgery, viral haemorrhagic fever (Ebola or Lassa virus) infection, blood donation, male health workers and an unfamiliar environment in terms of language and people. Such fears contributed to women returning home instead of travelling to the health centre they were referred to. Respondents identified three influential stakeholders who may potentially reduce fears around referral. The first stakeholder was the chief of the village or town, whose advice and instruction were of substantial influence on the women's and relatives' referral compliance.

For peer review only - http://bmjopen.bmj.com/site/about/guidelines.xhtml

"My chief is not staying that far from me. So sometimes I call him when there is a case, when there is need for him to come here, as stakeholder, that he can talk to this person." (male CHA, MCHP)

The second group of stakeholders, recognised by health workers as similarly influential, consisted of relatives in Freetown, the capital of Sierra Leone. One state-enrolled community health nurse (SECHN) stated:

"They will always listen to their relatives out there." (female SECHN, MCHP)

These relatives in Freetown were contacted and requested to attempt to convince the woman of the necessity of the referral and to adhere to the referral instructions. Finally, according to the health workers, women who had previously been referred to a district hospital and returned safely had a positive influence on a woman's perceptions regarding referrals to district hospitals.

4) Logistical constraints

Medicine shortage as a burden on the referral system. The logistical constraints of dealing with stockouts of medication in PHUs comprised an additional burden on the referral system. Many health workers, especially those working in CHCs, complained of struggling with medication shortages. Injectable antibiotics were often mentioned as insufficient for the purposed term.

"But mediciation, logistics, is much more paramount. We need IV fluids, we need drugs." (male CHA, CHC)

When medication stocks had been exhausted, the health worker was faced with two options. The first option was to request money from the woman to purchase medication at a

For peer review only

For peer review only - http://bmjopen.bmj.com/site/about/guidelines.xhtml

local pharmacy. One health worker described the friction this created with the Free Health Care Scheme for pregnant and lactating women, since it was by law illegal to request money from these women.

> *"I am not going to ask her to pay for the service I am rendering but just to provide the drug. But me that is punishable crime, I cannot… Then I be in fault."* (male CHA, CHC)

However, sending a woman directly to the pharmacy to buy medication themselves was not safe according to several health workers. They expressed their distrust in local pharmacists as they suspected them of not being properly trained and sometimes giving the wrong medication as well as administering injectable drugs themselves against regulations.

Health workers were therefore often forced to resort to referral to a district hospital in order for a woman to access the correct medication. The CHA voiced his desperation:

> *"So what would you do? You just have to refer."* (male CHA, CHC)

He also expressed his worry about the reaction from the district hospital after receiving such referrals. He feared that the district hospital would doubt the competency of the health workers at the PHU referring a woman who could potentially be managed at their own facility. Finally, some respondents pointed out that referring such a woman exposed them to additional adverse outcomes such as the relatives falling back on traditional medicine or going to a local pharmacy, since this was cheaper than paying for referral transportation.

Inadequate ambulance availability. Tonkolili District has a limited number of ambulances available for the transport of women with emergency obstetric complications. Many health workers complained about the fact that when they call for an ambulance, they are told that

For peer review only - http://bmjopen.bmj.com/site/about/guidelines.xhtml

the ambulance has broken down or to wait since the ambulance is on its way to a different, sometimes very distant PHU.

> *"If you call the ambulance, at times the ambulance takes three to four hours before arriving here."* (male CHO, CHC)

It was also noted that it sometimes takes a long time before the ambulance team, comprised of a driver and a nurse, is mobilised at the district hospital and the ambulance is finally under way. One CHA summarised the problem as:

> *"So sometimes it's very difficult; the time the ambulance is here, the patient is seriously in a critical condition."* (male CHA, CHC)

Another problem reported was the road accessibility of certain PHUs. Some of these PHUs can only be reached by motorbike and on foot. Accessibility is worse during rainy season. Ambulance transport during the rainy season was even stated to be not possible at all for several PHUs.

DISCUSSION

This study highlights several aspects of the obstetric referral system in rural Sierra Leone, which require attention in order to provide timely and adequate management of women with emergency obstetric complications.

Importance of communication between staff of different health centres

The importance of communication between health centres to achieve an effective referral system was widely acknowledged by participating providers. Health care workers generally concurred with each other on the advantages of receiving advice on whether to continue management, or to refer a woman instead. However, our findings concerning the practice of

For peer review only

waiting for a higher-cadre health worker from a different health facility to arrive and personally examine the woman before advising on a referral decision, are in disagreement with protocol and would increase type 2 delay. This delay can be largely abated by adequate use of mobile phones to communicate with higher-cadre health workers for advice regarding referral decisions [15], as well as further education for MCH aids who will not need a second opinion anymore. Furthermore, the reluctance of lower-cadre health workers in referring women directly to a district hospital and thereby bypassing the higher-cadre health worker's judgment as well as the reluctance of referring women to a CHC with the advice to further refer to a district hospital reveal potentially harmful hierarchy between health workers. From Tanzania, Ueno et al. reported a similar atmosphere of hierarchy and lack of cooperation between different cadres of health workers and levels of health facilities as a challenge to EmOC service delivery [16].

Our findings also imply that following referral, district hospitals need to take initiative in providing health workers in PHUs with feedback in order to improve and encourage future referrals and follow-up management of the woman after discharge from the district hospital. Studies in Ghana, Burundi and Northern Uganda reported similar demands for feedback after referral [17-19]. Multiple other studies have described related gaps in communication surrounding obstetric referrals [20, 21] and have specified the critical role of communication in an effective referral system [21-24]. Improvement in communication between health facilities and health workers is a necessary first step towards improving the referral system in northern Tonkolili District.

Underlying influences on decision-making

For peer review only

For peer review only - http://bmjopen.bmj.com/site/about/guidelines.xhtml

Our findings point at a mindset of some health workers regarding obstetric referrals that has potential adverse effects on timely management of women with emergency obstetric complications. Maternal mortality and morbidity were seen as tragic events for women and relatives, but also alarming for themselves as health workers. Referral and the ensuing transfer of responsibility was regarded as an option to prevent themselves from being blamed in case of a complication. It is hypothesised that this mind-set is an adverse result of the increased awareness of, and attention to, the high maternal mortality and morbidity rates in Sierra Leone. Obstetric audits have been proven to be an effective method of reducing maternal mortality and morbidity [25-27], but a negative impact on work satisfaction and motivation have also been reported [27, 28]. However, our findings provide limited evidence and further research in rural Sierra Leone is essential to accurately analyse this information.

Another underlying influence which became apparent throughout the interviews was the persuasiveness of women and relatives who did not want to be referred to a different health facility. Such persuasiveness has potential to delay the referral decision made by a health worker and thus results in phase 1 and phase 2 delays. Also, it may lead to over-confidence of lower-cadre health workers respecting their ability in managing women with obstetric complications, as has been previously reported in Sierra Leone by Theuring et al. [13].

Improving women's compliance with referral

Fears experienced by women, as reported by health workers, to accept referral to a district hospital such as fear of operations, blood donation, male health workers conducting births, and a new environment were largely similar to those previously reported by multiple studies in similar settings [17, 19, 29, 30]. A lingering fear of Ebola in district hospitals in post-Ebola

For peer review only

For peer review only - http://bmjopen.bmj.com/site/about/guidelines.xhtml

regions including Sierra Leone has been described by several other studies [13, 31, 32]. Three groups of influential stakeholders were identified: local village chiefs, relatives in the capital, Freetown; and women who have previously been referred to a district hospital and have returned safely. Consulting these stakeholders in case of referral refusal will potentially increase women's compliance. These stakeholders are understood to be similarly influential in all rural areas of Sierra Leone.

Logistical constraints

The influence of medication shortage in PHUs on the effectiveness of the referral system becomes apparent through our findings. The combined effect of the Free Health Care Scheme [33] and the distrust in local pharmacies forces health workers into avoidable referrals to district hospitals. In turn, these referrals lead women to a choice of options from traditional medication or buying medication from a local pharmacy. The shortage of medication in PHUs is a burden on and complicating factor of the referral system in northern Tonkolili District; however, adequate availability of medication should not avert mandatory referrals of women with obstetric complications, which require management in district hospitals.

The shortage of ambulances for transport of women from lower-level health facilities to district hospitals is a commonly reported contributor to phase 2 delay in sub-Saharan Africa [17-19, 34, 35]. Our findings show that this barrier to access to adequate emergency obstetric care is also present in rural Sierra Leone. The inability of ambulances to reach certain PHUs, due to arduous terrain such as steep hills and river crossings and poor road conditions, worsened by seasonal rains, displays the poor infrastructure of rural Sierra Leone.

For peer review only

For peer review only - http://bmjopen.bmj.com/site/about/guidelines.xhtml

In February 2019, after our data collection was completed, the Ministry of Health and Sanitation of Sierra Leone and the non-governmental organisation Doctors with Africa CUAMM launched the National Emergency Medical Service (NEMS), which provides free-of-charge ambulance service in all of Sierra Leone. The implementation of the NEMS allows for imperative reduction in delay in access to adequate emergency obstetric care in district hospitals. This reduction in delay is possible as the number of functioning ambulances is increased, and as the travel time is decreased as soon as the ambulances are not only stationed in district hospitals any longer, but also in PHUs.

Strengths and Limitations

A strength of this study is the selection of PHUs and health workers for the interviews, which is representative of health facilities and health workers in rural Sierra Leone. Therefore, interventions targeting the obstetric referral system in other areas of rural Sierra Leone can be supported by our findings. Another strength is the use of open-ended questions during the interviews, which allowed the participants to express their own experiences and feelings. Additionally, as this study was conducted before the implementation of the NEMS, this study allows for a follow-up study analysing the effect of the NEMS. The authors hypothesise that only the themes 'communication' and 'logistical constraints' have been affected by the NEMS.

A limitation of this study was that the data collected using semi-structured interviews with health workers were not triangulated with data from women and their relatives. However, results from previously conducted research concerning the perspectives of women and their relatives have been discussed to further validate our data. Additionally, a description of the

For peer review only

major reasons of referral would have been of added value to our manuscript. Lastly, the interviews were conducted in English while the participants were more familiar with Krio.

CONCLUSION

In the perspectives of health care workers, delay in access to adequate emergency obstetric care is caused by lack of communication between health workers at different facilities, lack of involvement of influential stakeholders, medication shortage and lack of ambulance services. Of note, fear among women and their relatives for them to be referred is another cause of delay. Furthermore, the decision-making of health care workers concerning referral is negatively influenced by an atmosphere of hierarchy and fear of having maternal deaths and other severe complications at their facility.

Interventions that may reduce delay in access to adequate emergency obstetric care include communication by mobile phones for advice regarding referral decisions and for feedback after a referral decision has been made. Involvement of influential stakeholders to increase women's compliance to referral is an additional intervention that may be considered. Additionally, consistent use of a standardised management protocol at the different levels of health facilities may reduce delay in access to emergency obstetric care.

This study highlights factors that may complicate timely and adequate referral of women in need of emergency obstetric care. As this delay is an underlying cause of the high MMR in rural Sierra Leone, these potential sources and causes of delay must be considered in efforts to reduce maternal mortality.

LIST OF ABBREVIATIONS

MMR: Maternal Mortality Ratio;

For peer review only

BEmONC: Basic Emergency Obstetric and Neonatal Care;

CEmONC: Comprehensive Emergency Obstetric and Neonatal Care;

MCHP: Maternal and Child Health Post;

CHP: Community Health Post;

CHC: Community Health Centre;

PHU: Peripheral Health Unit;

CHO: Community Health Officer;

CHA: Community Health Assistant;

SECHN: State-Enrolled Community Health Nurse;

MCH-aid: Maternal and Child Health Aid;

NEMS: National Emergency Medical Service

DECLARATIONS

Ethics approval and consent to participate

The study proposal was endorsed by the Masanga Medical Research Unit Scientific Review Committee. Ethical approval was obtained from the Sierra Leone Ethics and Scientific Review Committee. Permission to conduct the study in Tonkolili District was obtained from the District Health Management Team. Written informed consent was obtained from interview participants.

Data availability

For peer review only

The datasets used and/or analysed during the current study are available from the corresponding author on reasonable request.

Competing Interests

All authors declare that they have no competing interests.

Funding

This research received no specific grant from any funding agency in the public, commercial or not-for-profit sectors.

Author Contributions

RP carried out the field work, analysed the data and wrote the first draft of the paper. HM and JvN conceived the study and contributed to phrasing the study question, data interpretation and writing of the paper. AF, PSK and MPG contributed to data interpretation and writing of the paper. TvdA oversaw the conduct of the study and contributed to data interpretation and writing of the paper. All authors have contributed to the writing of, and approved the final version of the paper.

Acknowledgements

The authors wish to acknowledge all the staff in health care facilities that participated in the study.

REFERENCES

1. Statistics Sierra Leone (SSL) and ICF International. Sierra Leone Demographic and Health Survey 2013; Freetown, Sierra Leone and Rockville, Maryland, USA: SSL and ICF

For peer review only

International; 2014 [Available from: https://dhsprogram.com/pubs/pdf/fr297/fr297.pdf accessed February 2019.

2. United Nations. Sustainable Development Goals 2015 [Available from: https://www.un.org/sustainabledevelopment/sustainable-development-goals/ accessed July 1 2019.

3. Sierra Leone Ministry of Health and Sanitation. Sierra Leone National Reproductive, Maternal, Newborn, Child and Adolescent Health Strategy 2017-2021 [Available from: https://www.afro.who.int/publications/sierra-leone-national-reproductive-maternal-newborn-child-and-adolescent-health accessed February 2019.

4. Sochas L, Channon AA, Nam S. Counting indirect crisis-related deaths in the context of a low-resilience health system: the case of maternal and neonatal health during the Ebola epidemic in Sierra Leone. *Health Policy and Planning* 2017;32(Suppl 3):32-39. doi: 10.1093/heapol/czx108 [published Online First: 2017/11/18]

5. Huizenga E, van der Ende J, Zwinkels N, et al. A Modified Case Definition to Facilitate Essential Hospital Care During Ebola Outbreaks. *Clin Infect Dis* 2019;68(10):1763-68. doi: 10.1093/cid/ciy798 [published Online First: 2018/09/22]

6. Treacy L, Bolkan HA, Sagbakken M. Distance, accessibility and costs. Decision-making during childbirth in rural Sierra Leone: A qualitative study. *PLoS One* 2018;13(2):e0188280. doi: 10.1371/journal.pone.0188280 [published Online First: 2018/02/21]

7. Thaddeus S, Maine D. Too far to walk: maternal mortality in context. *Soc Sci Med* 1994;38(8):1091-110. [published Online First: 1994/04/01]

8. Geleto A, Chojenta C, Musa A, et al. Barriers to access and utilization of emergency obstetric care at health facilities in sub-Saharan Africa: a systematic review of

For peer review only

literature. *Syst Rev* 2018;7(1):183. doi: 10.1186/s13643-018-0842-2 [published Online First: 2018/11/15]

9. Tong A, Sainsbury P, Craig J. Consolidated criteria for reporting qualitative research (COREQ): a 32-item checklist for interviews and focus groups. *Int J Qual Health Care* 2007;19(6):349-57. doi: 10.1093/intqhc/mzm042 [published Online First: 2007/09/18]

10. Ameh C, Msuya S, Hofman J, et al. Status of emergency obstetric care in six developing countries five years before the MDG targets for maternal and newborn health. *PLoS One* 2012;7(12):e49938. doi: 10.1371/journal.pone.0049938 [published Online First: 2012/12/14]

11. Bailey PE, van Roosmalen J, Mola G, et al. Assisted vaginal delivery in low and middle income countries: an overview. *Bjog* 2017;124(9):1335-44. doi: 10.1111/1471-0528.14477 [published Online First: 2017/02/01]

12. Ministry of Health and Sanitation and UNFPA. Emergency Obstetric and Neonatal Care Guidelines.

13. Theuring S, Koroma AP, Harms G. "In the hospital, there will be nobody to pamper me": a qualitative assessment on barriers to facility-based delivery in post-Ebola Sierra Leone. *Reprod Health* 2018;15(1):155. doi: 10.1186/s12978-018-0601-9 [published Online First: 2018/09/17]

14. Malterud K. Systematic text condensation: a strategy for qualitative analysis. *Scand J Public Health* 2012;40(8):795-805. doi: 10.1177/1403494812465030 [published Online First: 2012/12/12]

For peer review only

15. Noordam AC, Kuepper BM, Stekelenburg J, et al. Improvement of maternal health services through the use of mobile phones. *Trop Med Int Health* 2011;16(5):622-6. doi: 10.1111/j.1365-3156.2011.02747.x [published Online First: 2011/02/24]

16. Ueno E, Adegoke AA, Masenga G, et al. Skilled birth attendants in Tanzania: a descriptive study of cadres and emergency obstetric care signal functions performed. *Matern Child Health J* 2015;19(1):155-69. doi: 10.1007/s10995-014-1506-z [published Online First: 2014/05/06]

17. Nwameme AU, Phillips JF, Adongo PB. Compliance with emergency obstetric care referrals among pregnant women in an urban informal settlement of Accra, Ghana. *Matern Child Health J* 2014;18(6):1403-12. doi: 10.1007/s10995-013-1380-0 [published Online First: 2013/11/02]

18. Chi PC, Bulage P, Urdal H, et al. Barriers in the Delivery of Emergency Obstetric and Neonatal Care in Post-Conflict Africa: Qualitative Case Studies of Burundi and Northern Uganda. *PLoS One* 2015;10(9):e0139120. doi: 10.1371/journal.pone.0139120 [published Online First: 2015/09/26]

19. Afari H, Hirschhorn LR, Michaelis A, et al. Quality improvement in emergency obstetric referrals: qualitative study of provider perspectives in Assin North District, Ghana. *BMJ Open* 2014;4(5):e005052. doi: 10.1136/bmjopen-2014-005052 [published Online First: 2014/05/17]

20. Austin A, Gulema H, Belizan M, et al. Barriers to providing quality emergency obstetric care in Addis Ababa, Ethiopia: Healthcare providers' perspectives on training, referrals and supervision, a mixed methods study. *BMC Pregnancy Childbirth* 2015;15:74. doi: 10.1186/s12884-015-0493-4 [published Online First: 2015/04/18]

For peer review only - http://bmjopen.bmj.com/site/about/guidelines.xhtml

21. Holmes W, Kennedy E. Reaching emergency obstetric care: overcoming the 'second delay'. Melbourne, Australia: Burnet Institute on behalf of Compass, the Women's and Children's Health Knowledge Hub, 2010.

22. Tayler-Smith K, Zachariah R, Manzi M, et al. An ambulance referral network improves access to emergency obstetric and neonatal care in a district of rural Burundi with high maternal mortality. *Trop Med Int Health* 2013;18(8):993-1001. doi: 10.1111/tmi.12121 [published Online First: 2013/05/21]

23. Tayler-Smith K, Zachariah R, Manzi M, et al. Achieving the millennium development goal of reducing maternal mortality in rural Africa: an experience from Burundi. *Trop Med Int Health* 2013;18(2):166-74. doi: 10.1111/tmi.12022 [published Online First: 2012/11/21]

24. Fournier P, Dumont A, Tourigny C, et al. Improved access to comprehensive emergency obstetric care and its effect on institutional maternal mortality in rural Mali. *Bull World Health Organ* 2009;87(1):30-8. [published Online First: 2009/02/07]

25. van den Akker T, Mwagomba B, Irlam J, et al. Using audits to reduce the incidence of uterine rupture in a Malawian district hospital. *Int J Gynaecol Obstet* 2009;107(3):289-94. doi: 10.1016/j.ijgo.2009.09.005 [published Online First: 2009/10/23]

26. van den Akker T, van Rhenen J, Mwagomba B, et al. Reduction of severe acute maternal morbidity and maternal mortality in Thyolo District, Malawi: the impact of obstetric audit. *PLoS One* 2011;6(6):e20776. doi: 10.1371/journal.pone.0020776 [published Online First: 2011/06/17]

For peer review only - http://bmjopen.bmj.com/site/about/guidelines.xhtml

27. Ivers N, Jamtvedt G, Flottorp S, et al. Audit and feedback: effects on professional practice and healthcare outcomes. *Cochrane Database Syst Rev* 2012(6):Cd000259. doi: 10.1002/14651858.CD000259.pub3 [published Online First: 2012/06/15]

28. Bakker W, van den Akker T, Mwagomba B, et al. Health workers' perceptions of obstetric critical incident audit in Thyolo District, Malawi. *Trop Med Int Health* 2011;16(10):1243-50. doi: 10.1111/j.1365-3156.2011.02832.x [published Online First: 2011/07/20]

29. Wright K, Banke-Thomas A, Sonoiki O, et al. Opinion of women on emergency obstetric care provided in public facilities in Lagos, Nigeria: A qualitative study. *Health Care Women Int* 2017;38(6):527-43. doi: 10.1080/07399332.2016.1234482 [published Online First: 2016/09/10]

30. Liambila WN, Kuria SN. Birth attendance and magnitude of obstetric complications in Western Kenya: a retrospective case-control study. *BMC Pregnancy Childbirth* 2014;14:311. doi: 10.1186/1471-2393-14-311 [published Online First: 2014/09/10]

31. Van Bortel T, Basnayake A, Wurie F, et al. Psychosocial effects of an Ebola outbreak at individual, community and international levels. *Bull World Health Organ* 2016;94(3):210-4. doi: 10.2471/blt.15.158543 [published Online First: 2016/03/12]

32. Jones T, Ho L, Kun KK, et al. Rebuilding people-centred maternal health services in post-Ebola Liberia through participatory action research. *Glob Public Health* 2018;13(11):1650-69. doi: 10.1080/17441692.2018.1427772 [published Online First: 2018/02/01]

33. Wakabi W. Mothers and infants to get free health care in Sierra Leone. *Lancet* 2010;375(9718):882. doi: 10.1016/s0140-6736(10)60371-6 [published Online First: 2010/03/17]

For peer review only

34. Mirkuzie AH, Sisay MM, Reta AT, et al. Current evidence on basic emergency obstetric and newborn care services in Addis Ababa, Ethiopia; a cross sectional study. *BMC Pregnancy Childbirth* 2014;14:354. doi: 10.1186/1471-2393-14-354 [published Online First: 2014/10/11]

35. Echoka E, Makokha A, Dubourg D, et al. Barriers to emergency obstetric care services: accounts of survivors of life threatening obstetric complications in Malindi District, Kenya. *Pan Afr Med J* 2014;17 Suppl 1:4. doi: 10.11694/pamj.supp.2014.17.1.3042 [published Online First: 2014/03/20]

For peer review only

For peer review only - http://bmjopen.bmj.com/site/about/guidelines.xhtml

SUPPLEMENTARY FILE 1

COREQ checklist of reporting

Topic	Item No.	Guide Questions/Description	Reported on Page No.
**Domain 1: Research team and reflexivity**			
Personal characteristics			
Interviewer/facilitator	1	Which author/s conducted the interview or focus group?	8
Credentials	2	What were the researcher's credentials? E.g. PhD, MD	8
Occupation	3	What was their occupation at the time of the study?	8
Gender	4	Was the researcher male or female?	8
Experience and training	5	What experience or training did the researcher have?	8
Relationship with participants			
Relationship established	6	Was a relationship established prior to study commencement?	10
Participant knowledge of the interviewer	7	What did the participants know about the researcher? e.g. personal goals, reasons for doing the research	10
Interviewer characteristics	8	What characteristics were reported about the inter viewer/facilitator? e.g. Bias, assumptions, reasons and interests in the research topic	10
**Domain 2: Study design**			
Theoretical framework			
Methodological orientation and Theory	9	What methodological orientation was stated to underpin the study? e.g. grounded theory, discourse analysis, ethnography, phenomenology, content analysis	12
Participant selection			
Sampling	10	How were participants selected? e.g. purposive, convenience, consecutive, snowball	9, 10
Method of approach	11	How were participants approached? e.g. face-to-face, telephone, mail, email	10
Sample size	12	How many participants were in the study?	10
Non-participation	13	How many people refused to participate or dropped out? Reasons?	10
Setting			
Setting of data collection	14	Where was the data collected? e.g. home, clinic, workplace	8, 10, 11
Presence of non-participants	15	Was anyone else present besides the participants and researchers?	11
Description of sample	16	What are the important characteristics of the sample? e.g. demographic data, date	6, 10
Data collection			
Interview guide	17	Were questions, prompts, guides provided by the authors? Was it pilot tested?	11
Repeat interviews	18	Were repeat inter views carried out? If yes, how many?	11
Audio/visual recording	19	Did the research use audio or visual recording to collect the data?	11
Field notes	20	Were field notes made during and/or after the interview or focus group?	N/A
Duration	21	What was the duration of the inter views or focus group?	11
Data saturation	22	Was data saturation discussed?	N/A
Transcripts returned	23	Were transcripts returned to participants for comment and/or correction?	11

For peer review only

Topic	Item No.	Guide Questions/Description	Reported on Page No.
**Domain 3: analysis and findings**			
Data analysis			
Number of data coders	24	How many data coders coded the data?	12
Description of the coding tree	25	Did authors provide a description of the coding tree?	12
Derivation of themes	26	Were themes identified in advance or derived from the data?	12
Software	27	What software, if applicable, was used to manage the data?	12
Participant checking	28	Did participants provide feedback on the findings?	11
Reporting			
Quotations presented	29	Were participant quotations presented to illustrate the themes/findings? Was each quotation identified? e.g. participant number	13-19
Data and findings consistent	30	Was there consistency between the data presented and the findings?	24
Clarity of major themes	31	Were major themes clearly presented in the findings?	13
Clarity of minor themes	32	Is there a description of diverse cases or discussion of minor themes?	N/A

Developed from: Tong A, Sainsbury P, Craig J. Consolidated criteria for reporting qualitative research (COREQ): a 32-item checklist for interviews and focus groups. *International Journal for Quality in Health Care*. 2007. Volume 19, Number 6: pp. 349 – 357

For peer review only

SUPPLEMENTARY FILE 2

Signal functions of BEmONC and CEmONC

Basic Emergency Obstetric and Neonatal Care (BEmONC)

1. Parenteral treatment of infections (antibiotics)
2. Parenteral treatment of severe pre-eclampsia/eclampsia (e.g., $MgSO_4$)
3. Treatment of PPH (e.g., uterotonics)
4. Manual vacuum aspiration of retained products of conception
5. Assisted vaginal delivery (e.g., vacuum-assisted delivery)
6. Manual removal of placenta
7. New-born resuscitation

Comprehensive Emergency Obstetric and Neonatal Care (CEmONC)

All components of BEmONC, plus

1. Surgical capability, including anaesthesia (e.g., Caesarean Section)
2. Blood transfusion

For peer review only

SUPPLEMENTARY FILE 3

Interview Guide Obstetric Referral System Northern Tonkolili District

General

1. Describe the catchment area of this health facility (number of villages, terrain).
2. Describe this health facility (number and competency of staff, average number of births and referrals).
3. Describe the referral options of this health facility (nearest health facility, nearest CHC, nearest district hospital).

Authorisation

4. Which of the staff is allowed to decide to refer women to another facility?
5. Is there always staff present that is allowed to independently refer women?
6. Does the CHO or midwife in the CHC have to be contacted before referring a woman to another facility?

Referral Process

7. Describe the steps taken when referring a pregnant or post-partum women.
8. Describe how/if referral notes are used in this health facility.
9. Describe the benefit of referral notes.
10. Describe how/if feedback is received from the referral facility.
11. Describe how/if advice is obtained from other health workers before referral.
12. Describe how/if national guidelines are used for referring a woman.
13. Describe how/if maternity patients without an emergency indication are referred to a different health facility.

Accessibility

14. Describe the accessibility of Magburaka Government Hospital from here.
15. Describe the accessibility of Masanga Hospital from here.
16. If you were to refer 10 women to a hospital, how many of them do you think will actually arrive at the hospital?
17. Describe some reasons why referred women sometimes refuse referral.
18. Describe transportation options available for referral.

Ambulance Referrals

19. Describe the process of ordering an ambulance from Magburaka Government Hospital.
20. Describe the process of ordering an ambulance from Masanga Hospital.
21. How many times have you ordered an ambulance in the past month?
22. How long does it take for the ambulance to arrive at your health facility after you have ordered the ambulance? (from Magburaka/Masanga)
23. Which of the staff is allowed to order an ambulance?
24. Is there always staff present that is allowed to order an ambulance?

For peer review only

25. Does the CHO or midwife in the CHC have to be contacted before ordering an ambulance?
26. Is transferral by ambulance free for the woman?
27. Describe problems with the current ambulance referral system.
28. Describe recommendations for improving the ambulance referral system.

ANC/under 5

29. Describe the antenatal care in this health facility (frequency of ANC-days, number of women)
30. Describe how/if governmental Maternity Record Cards are used during ANC visits, delivery and postnatal checks.

Masanga Hospital

31. Describe recommendations for improving the health care at Masanga Hospital.
32. Is Masanga Hospital an official referral hospital in Tonkolili District?
33. Is healthcare free for pregnant and lactating women and children under 5 at Masanga Hospital?

For peer review only

SUPPLEMENTARY FILE 4

Original study protocol

Includes protocol regarding quantitative research which is not included in submitted article

For peer review only

For peer review only - http://bmjopen.bmj.com/site/about/guidelines.xhtml

The current situation of maternal health in Tonkolili District and the perceptions of health workers at peripheral health units in the catchment area of Masanga Hospital concerning the current referral system of obstetric patients

For peer review only

Lead applicant

Ryan Proos, medical student Leiden University Medical Center, Masanga Hospital Sierra Leone

Correspondence address: e-mail: rmjproos@gmail.com

Supervisor

Dr. T. van den Akker, Leiden University Medical Center, Netherlands (Gynaecology and Obstetrics)

Research team

Hanna Mathéron, MD Global Health and Tropical Medicine, Medical Officer Masanga Hospital

Background

Sierra Leone is one of the most dangerous countries for women to be pregnant and to give birth. According to the Sierra Leone Demographic Health Survey (2013)[1], Sierra Leone has a maternal mortality ratio (MMR) of 1,165 per 100,000 live births, the highest in the world and more than six times the global average. The UN Sustainable Development Goals (SDGs), adopted in 2015, aim for a reduction of the global maternal mortality ratio to less than 70 per 100,000 live births in 2030. In line with these SDGs, the Ministry of Health and Sanitation of Sierra Leone launched the Reproductive, Maternal, Newborn, Child and Adolescent Health (RMNCAH) Strategy in 2017, which aims for a reduction of preventable deaths of women, children and adolescents and ensuring their well-being[2]. The target is to reduce the MMR of 1,165 per 100.000 live births to 650 per 100.000 live births by 2021. With launching this strategy, the government acknowledges the need for change, but there is still a long way to go to achieve this goal.

Masanga Hospital is situated in Tonkolili district, centrally located in the Northern Province of Sierra Leone. The catchment area of Masanga Hospital comprises of approximately 150,000 inhabitants and 15 peripheral health units (PHUs). Besides Masanga Hospital there are two other hospitals in Tonkolili district, Magburaka Hospital and Lion Heart Hospital in Yele.

Currently, there is no complete and trustworthy overview of maternal health in Tonkolili District. Therefore, a baseline assessment using the WHO Reproductive Health Indicators and the RMNCAH coverage targets is needed to work towards achieving the goal set by the Ministry.

It is hypothesized that the poor referral system is a major causative factor of the current poor maternal health situation in Tonkolili district. Perceptions of health workers at PHUs concerning the referral system of obstetric patients is necessary to identify areas of improvement.

Aims and Objectives

The aim of this research project is to do a retrospective, quantitative descriptive analysis of data collected during project to describe maternal health demography, geography, delivery and complication rates of Tonkolili district, especially in relationship to the catchment area of Masanga Hospital. Additionally, an assessment of health workers' perceptions of the current referral system of obstetric patients will be undertaken.

For peer review only

Research Questions

1. What is the current maternal health situation in Tonkolili District, Sierra Leone, in terms of WHO Reproductive Health Indicators and the RMNCAH coverage target matches?

The baseline assessment shall provide an overview of several WHO Reproductive Health Indicators and RMNCAH coverage targets in Tonkolili district over the period 2016-2018**.** Data collection will be done in the Peripheral Health Units (PHUs) and the district hospitals (Masanga Hospital, Magburaka Hospital and Lion Heart Hospital, Yele), in collaboration with the District Health Sister (DHS1), District Medical Officer (DMO) and the District Health Information System (DHIS).

The following data will be collected per health facility:

- Level of service provision (MCHP, CHP, CHC, District Hospital)
- Location in the catchment area
- Number of deliveries (spontaneous, assisted, CS)
- Number of live births
- Number of still births
- Number of maternal deaths
- Number of complications (antepartum haemorrhage (APH), postpartum haemorrhage (PPH), pregnancy-induced hypertension (PIH), pre-eclampsia, eclampsia, obstructed labour, post-partum sepsis)
- Number of referrals, reasons for referral
- Number of antenatal care visits
- Number of intermittent preventive treatment (IPT) during ANC

2. What are the perspectives of health workers at peripheral health units in the catchment area of Masanga Hospital concerning the current referral system of women with obstetric complications?

Perspectives of health workers working in PHUs in the catchment area of Masanga Hospital will be explored in several interviews.

The discussions will focus on the following topics:

- Communication between health centres
- Determinants of referral decision
- Actions taken before and during referral
- Effect of antenatal care on referrals
- Referral constraints

For peer review only - http://bmjopen.bmj.com/site/about/guidelines.xhtml

Study Design

This research project is a retrospective, quantitative descriptive analysis. Additionally, a qualitative analysis will be done using interviews. Data collection will be done in collaboration with the District Health Sister (DHS1), District Medical Officer (DMO) and the DHIS.

Patients/Participants

All women that visited a PHU or hospital in Tonkolili district for antenatal care visits, health facility deliveries or pregnancy or delivery complications during the period 2016-2018 will be included in the baseline overview. Selection bias must be considered as not all pregnant women visit a health facility. Reproductive health data is available through the centralized District Health Information System. Access to the District Health Information System will be officially requested. Data is available of approximately 12,000 facility-based live births in the district per year and therefore roughly 36,000 facility-based live births in the specified period. However, it is estimated that 46% of pregnant women in Sierra Leone give birth at home[2]. There is therefore no data available of these homebirths, but these women might have visited a health facility for antenatal care or for post-partum complications.

Health workers at PHUs in the catchment area of Masanga Hospital will be asked to participate in the interviews focusing on their perceptions of the current obstetric referral system. Written informed consent will be obtained using the attached form. An interview guide will be used.

Statistical Methods

After centralization of the data in SPSS, a baseline overview of the variables named for research question 1 will be made for each PHU and hospital and for Tonkolili district. The qualitative data of the group discussions will be analyzed using systematic text condensation.

For peer review only

Results

The baseline assessment shall provide an overview of several WHO Reproductive Health Indicators and the RMNCAH coverage targets in Tonkolili district over the period 2016-2018. Regional differences in maternal health outcomes in Tonkolili District will be identified. These regional differences will be linked to the accessibility of emergency obstetric care in those regions. This baseline overview will provide a basis for further research and implementation of new interventions, aimed at reaching the goal set by the Ministry.

The perceptions of health workers at PHUs in the catchment area of Masanga Hospital will illuminate constraints of the current referral system of obstetric patients. Additionally, the quality of communication between different levels of health facilities concerning referrals will be described. Possible solutions as suggested by the health workers will be described.

The results will be summarized in a descriptive article.

For peer review only

For peer review only - http://bmjopen.bmj.com/site/about/guidelines.xhtml

Ethical Considerations

Ethical Approval

Ethical approval will be obtained from the Sierra Leone Ethics and Scientific Review Committee.

Selection of study population and recruitment of research participants

All patients registered at PHUs and hospitals in Tonkolili District for antenatal care visits, deliveries, and pregnancy complications in the period 2016-2018 are selected and included. Patients are free to refuse participation. This refutation must be apparent from the District Health Information System.

Informed consent process

Receiving medical care at a PHU or hospital implicitly indicates given consent to use of registered data for medical research through the District Health Information System. Written informed consent will be obtained from participants in the group discussions. The informed consent form is included in this application.

Risks of participation

Participation in the baseline overview exposes patients to minimal chance of harm. Participation does not include any clinical intervention. Confidentiality and anonymity of patient data is maximally safeguarded.

Participation in the group discussions exposes the health workers to minimal chance of harm. Confidentiality and anonymity of participant data is maximally safeguarded.

Inducements, financial benefits, and financial costs

Participation is not rewarded with any financial, material or healthcare service benefit.

Protection of research participants' privacy and confidentiality

Research participant data is centrally stored in the District Health Information System. This digital system is password protected. To ensure confidentiality, this data is only accessible by the head researcher and supervisors.

Group discussion participant data is stored on password-protected computers in Masanga Hospital. Only the head researcher has access to the names, functions and health facilities of the participants. Reported data is anonymous and cannot be traced back to the specific health facility.

Independence of research and conflicts of interests

Research is conducted on behalf of Masanga Hospital and in collaboration with Tonkolili District. No other parties are involved. The researchers certify that they have no conflicts of interests.

For peer review only

Schedule

1-8 weeks:

- Contact with DMO and DHS, attending the district meetings and the district maternal mortality meetings in Magburaka
- Application for funding
- Application for District Health Information System access

8-20 weeks

- Analysation of DHIS data
- Group discussions

20-28 weeks

- Analysation and illustration of DHIS data
- Writing descriptive article

GANTT chart

	W1-4	W5-8	W9-12	W13-16	W17-20	W20-24	W24-28
**DMO/DHS contact**							
**Initiation visits in PHUs/Hospitals**							
**DHIS access application**							
**Group discussions**							
**Data analysis**							
**Writing**							
**Submitting article**							

For peer review only

References

1. Sierra Leone Demographic and Health Survey 2013 via the DHS Program STATcompiler. (http://www.statcompiler.com).
2. Sierra Leone Reproductive, Maternal, Newborn, Child and Adolescent Health (RMNCAH) strategy 2017-2021, Ministry of Health Sierra Leone

For peer review only

For peer review only - http://bmjopen.bmj.com/site/about/guidelines.xhtml

MASANGA RESEARCH

Participant Informed Consent Form

Study title: Perceptions of health workers at peripheral health units in the catchment area of Masanga Hospital concerning the current referral system of obstetric patients

Researcher: Ryan Proos E-mail: ryanproos9@gmail.com

Institute: Masanga Medical Research Unit, Sierra Leone

	Add your initials if you agree
I agree that this interview will be recorded and that the data will be analyzed and reported anonymously.	
I understand that I can withdraw from this study at any time, without given reason and all my data will be deleted.	
I understand I can contact the Sierra Leone Ethics and Scientific Review Committee at any time (contact details below*).	

Name of participant: ……………………………………………………………………………….

Signature: ……………………………………………… Date: ……………………………….

Name of person taking consent: ……………………………………………………………….

Signature: ……………………………………………… Date: ……………………………….

* Contact details Sierra Leone Ethics and Scientific Review Committee:

E-mail: efoday@health.gov.sl

** this form needs to be signed and dated in presence of the patient. If preferred, the patient will receive a copy of this form. The forms will be stored in a secure location, ensuring patients privacy.

For peer review only - http://bmjopen.bmj.com/site/about/guidelines.xhtml
